# Meta-analysis of the efficacy of external application of Chinese medicine in the treatment of lower limb motor impairment in patients with post-stroke hemiplegia

**DOI:** 10.3389/fneur.2025.1691805

**Published:** 2025-11-28

**Authors:** Jingwen Zhang, Xingyu Kang, Jiajie Niu, Jingwen Zhao, Siyu Chen, Shuai Shi

**Affiliations:** 1 Heilongjiang University of Chinese Medicine, Harbin, Heilongjiang, China; 2 The Second Affiliated Hospital of Heilongjiang University of Chinese Medicine, Harbin, Heilongjiang, China

**Keywords:** external use of traditional Chinese medicine, hemiplegia, clinical efficacy, mechanism of action, systematic review

## Abstract

**Objective:**

To systematically evaluate the efficacy of external application of traditional Chinese medicine (TCM)—including TCM plaster application, TCM iontophoresis, hot compress, fumigation, fuming and washing, soaking, hot ironing, and cold compress—in the treatment of lower limb dysfunction in patients with post-stroke hemiplegia.

**Methods:**

We performed a comprehensive computer-based search of multiple databases (for domestic literature, China National Knowledge Infrastructure (CNKI), China Biomedical Literature Service System (CBM), Chongqing VIP Information Co., Ltd. (VIP), and Wanfang Database were used). The retrieval time limit was from the establishment of each database to the present. All randomized controlled trials (RCTs) and clinical observations evaluating the effect of TCM external application on lower limb motor function in post-stroke hemiplegia patients were collected. The literatures meeting the inclusion criteria were screened out. Two reviewers independently evaluated the methodological quality of the included studies and extracted effective data. The Cochrane bias risk assessment tool was used to evaluate the literature quality, and RevMan 5.4 software was used for meta-analysis.

**Results:**

A total of 43 RCTs involving 4,186 patients were finally included. The results of meta-analysis showed that TCM external application was significantly more effective than simple rehabilitation or recovery training in improving lower limb dysfunction after stroke, with notable improvements in motor function (e.g., Fugl-Meyer Assessment) and activities of daily living scores.

**Conclusion:**

External application of traditional Chinese medicine has significant efficacy and safety in the treatment of lower limb dysfunction after stroke. Potential mechanisms of action are also discussed.

## 1 Introduction

Stroke refers to a group of diseases caused by the rupture or obstruction of cerebral blood vessels, leading to brain tissue damage and subsequent impairment of the central nervous system. It represents the second leading cause of disability and mortality worldwide (1). Stroke has become a global problem that seriously jeopardizes human health, and it is characterized by high morbidity, high mortality, high disability, and high recurrence rate. Clinically, approximately two-thirds of stroke patients survive, but half of these survivors experience varying degrees of physical dysfunction (2), and most of them have motor dysfunction, mainly hemiplegia, and are often accompanied by cognitive, speech, swallowing, urinary incontinence, and psychological disorders, which seriously affects the patients’ ability to take care of themselves and their work ability, and reduces their quality of life (3). It causes serious harm to the daily lives of patients. Lower limb motor function is crucial for patients to be able to walk independently and carry out daily activities normally, so improving the lower limb motor function of hemiplegic patients after stroke is one of the key goals of rehabilitation therapy.

In the book “LiYuePianWen,” it is mentioned that “the theory of external treatment is the theory of internal treatment, and the medicine of external treatment is the medicine of internal treatment, and the difference is the law. There is no difference in the method of healing, but the method is magical.” This highlights that external and internal treatments in TCM share the same theoretical foundation, differing primarily in their routes of administration (4). The external use of Chinese medicine, such as Chinese medicine fumigation, Chinese medicine wet heat compress, Chinese medicine patch, etc., occupies an important position in traditional Chinese medicine therapies. These therapies are administered through the skin, mucous membrane, and other routes, aiming to promote local blood circulation, relieve muscle tension, improve nerve function, etc., and thus may have some therapeutic effects on post-stroke hemiparesis. In recent years, the external treatment of traditional Chinese medicine has demonstrated unique advantages in the treatment of post-stroke hemiplegia and has become a research hotspot. Thus, a meta-analysis to systematically evaluate the efficacy of TCM external therapy for lower limb dysfunction in this population is of considerable significance.

Although several studies have investigated the effects of TCM external therapies on lower limb function in post-stroke hemiplegia, the existing literature exhibits notable limitations: First, methodological quality varies considerably, with most randomized controlled trials featuring small sample sizes and inadequate reporting of key methodological details, such as random sequence generation, allocation concealment, and blinding. Second, interventions lack standardized protocols across studies, with significant variations in key parameters, such as herbal composition, formulation concentration, treatment duration, and frequency, leading to high result heterogeneity and difficulty in direct comparisons. Additionally, previous systematic reviews have predominantly focused on single therapies or single outcome measures, lacking integrated analyses of multidimensional indicators including lower limb motor function, activities of daily living, neurological deficits, and serum biomarkers. Therefore, this study aims to comprehensively evaluate the efficacy of traditional Chinese medicine external therapies on lower limb motor function in stroke-induced hemiplegia patients through systematic review and meta-analysis, while exploring potential mechanisms of action. This study represents the first systematic integration of Chinese and English literature in this field, concurrently analyzing outcomes, including lower limb motor function (Fugl-Meyer Assessment, FMA), activities of daily living (Barthel Index, Modified Barthel Index), neurological deficits (National Institutes of Health Stroke Scale, NIHSS), balance function (Berg Balance Scale, BBS), and serum biomarkers reflecting the fibrinolytic system (PAI-1, t-PA). This aims to provide a more comprehensive and reliable evidence base than previous studies.

## 2 Literature inclusion and exclusion criteria

### 2.1 Study registration

This systematic review was conducted and reported in accordance with international guidelines for meta-analyses. The study protocol was prospectively registered on the PROSPERO international prospective register of systematic reviews (Registration Number: CRD42024616854; Registration Link: https://www.crd.york.ac.uk/prospero/display_record.php?RecordID=616854). The implementation of this review strictly adhered to the pre-registered protocol, with no deviations. As this research synthesized data from previously published studies, it did not involve direct interaction with human or animal subjects or access to confidential personal data; therefore, it was exempt from institutional ethics committee approval. No funding was received for this study.

### 2.2 Inclusion criteria

Literature inclusion followed the PICOS principle, as detailed in Table 1.

**Table 1:** PICOS strategy—inclusion criteria.

PICOS	Inclusion criteria
Population	Patients diagnosed with stroke according to internationally recognized criteria, confirmed by CT or MRI; stable vital signs, conscious, with lower limb motor dysfunction; inclusion was not limited by gender, age, or race.
Interventions	The experimental group received TCM external therapy alone (e.g., TCM plaster, iontophoresis, hot compress, fumigation, soaking, hot ironing, and cold compress) or in combination with control group treatments.
Comparison	The control group implemented conventional treatment, rehabilitation training, or acupuncture intervention;
Outcome	There should be clear outcome indicators and quantitative values, and any one of the following outcome indicators can be included: total effective rate of treatment (total effective rate of treatment = number of cases (cured + effective + effective) / total number of cases × 100%), cure rate (cure rate of treatment = number of cured cases / total number of cases × 100%), and evaluation of motor function and spasticity status of the scale, such as the Fugl-meyer Limb Movement Rating Scale (Fugl-meyer). Functional rating scale (Fugl-meyer assessment scale, FMA), modified Ashworth scale (modified Ashworth scale, MAS), unarmed muscular strength assessment (MMT) grading, daily living activities scores: Barthel index, modified Barthel index, ADL score; neurological deficit score: National Institutes of Health Stroke Scale (NIHSS), balance function: Berg Balance Scale (BBS), and other rehabilitation assessment indexes.
Study design	Randomized clinical trials (RCTs), language restricted to Chinese and English.

CT: Computed tomography; MRI: Magnetic resonance imaging.

### 2.3 Exclusion criteria

(1) Hemiplegia due to hemorrhagic stroke and other diseases;
(2) Dissertations, case reports, conference reports, and reviews;
(3) Literature that was not available in full text, had incomplete primary data, or could not be extracted or converted for acquisition;
(4) Interventions where outcome indicators were not met;
(5) Studies without a control group or with multiple control groups;
(6) Studies with a sample size of fewer than 30 participants were excluded. This threshold was set to mitigate the risk of bias associated with small-scale studies, enhance the statistical stability of the pooled results, and ensure the included trials possessed a minimum level of clinical relevance and feasibility.
(7) Studies with unknown diagnostic criteria and incorrect data.

### 2.4 Literature search strategy

Several Chinese and English databases were searched by computer, and CNKI, China Biomedical Literature Service (CBM), Wipo, and Wanfang databases were used for literature collection in Chinese, while Pubmed, Embase, Web of Science, and Cochrane Library were used for foreign literature collection, to collect information about the intervention of Chinese herbal medicines in hemiplegia after stroke. Randomized controlled trials (RCTs) on the intervention of external use of traditional Chinese medicine in lower limb dysfunction in patients with hemiplegia after stroke were collected. The search timeframe was usually from database construction to 15 October 2024 to ensure inclusion of the most recent research results. To identify studies relevant to the purpose of the meta-analysis, we searched using a comprehensive set of search terms, including (1) “stroke” or “cerebrovascular accident” or “cerebral stroke” or “Cerebral infarction” or “Cerebral embolism” or “Cerebral ischemia” or “Ischemic encephalopathy,” (2) “hemiplegia” or “lower extremity muscle strength” or “muscle tone,” and (3) “External application of traditional Chinese medicine” or “patch” or “fumigation” or “Foaming” or “Hot ironing.” The search terms were based on keywords rather than MeSH terms to increase the sensitivity of the database search. However, comparisons were made with MeSH search terms before the database search to ensure that all relevant MeSH search terms were included in the search. Retrieval formulations were developed using logical characters, wildcards, range operators, etc. Taking the PubMed database as an example, the detailed search strategy is shown in Table 2.

**Table 2:** PubMed search strategy.

#1	Hemiplegia OR Hemiplegias OR Monoplegia OR Monoplegias OR Hemiplegia, Crossed OR Crossed Hemiplegia OR Crossed Hemiplegias OR Hemiplegias, Crossed OR Hemiplegia, Flaccid OR Flaccid Hemiplegia OR Flaccid Hemiplegias OR Hemiplegias, Flaccid OR Hemiplegia, Infantile OR Hemiplegias, Infantile OR Infantile Hemiplegia OR Infantile Hemiplegias OR Hemiplegia, Post-Ictal OR Hemiplegia, Post Ictal OR Hemiplegias, Post-Ictal OR Post-Ictal Hemiplegia OR Post-Ictal Hemiplegias OR Hemiplegia, Spastic OR Hemiplegias, Spastic OR Spastic Hemiplegia OR Spastic Hemiplegias OR Hemiplegia, Transient OR Hemiplegias, Transient OR Transient Hemiplegia OR Transient Hemiplegias
#2	Stroke OR Cerebrovascular Accident OR Cerebrovascular Accidents OR Cerebral Stroke OR Cerebral Strokes OR Stroke, Cerebral OR Strokes, Cerebral OR Cerebrovascular Apoplexy OR Apoplexy, Cerebrovascular OR Vascular Accident, Brain OR Brain Vascular Accident OR Brain Vascular Accidents OR Vascular Accidents, Brain OR Cerebrovascular Stroke OR Cerebrovascular Strokes OR Stroke, Cerebrovascular OR Strokes, Cerebrovascular OR Apoplexy OR CVA OR CVAs OR Stroke, Acute OR Acute Stroke OR Acute Strokes OR Strokes, Acute OR Cerebrovascular Accident, Acute OR Acute Cerebrovascular Accident OR Acute Cerebrovascular Accidents OR Cerebrovascular Accidents, Acute
#3	External treatment of traditional Chinese medicine OR local rubbing OR sticking OR fumigation OR Chinese medicine introduction OR washing OR hot ironing OR cold compress
#4	#1AND#2AND#3

### 2.5 Study quality evaluation and data extraction

Two researchers independently executed the search strategy and screened the retrieved literature against inclusion and exclusion criteria. The retrieved literature was imported into EndNote 20 for management and duplicate removal. Following the screening of titles and abstracts against the inclusion and exclusion criteria, studies with sample sizes below our pre-specified threshold (*n* < 30) were excluded for the reasons stated in Section 2.3 (6). The remaining literature was further screened by reading the full text of the literature, and the screened literature was finally compared. The methodological quality of the included randomized controlled trials (RCTs) was assessed using the Cochrane Risk of Bias tool for Randomized Trials (RoB 2.0). Two reviewers independently evaluated each study across five domains: (1) bias arising from the randomization process; (2) bias due to deviations from the intended interventions; (3) bias due to missing outcome data; (4) bias in measurement of the outcome; and (5) bias in selection of the reported result. Specific criteria for judgment within each domain were predefined according to the RoB 2.0 guidance. Discrepancies in assessment were resolved through discussion until consensus was reached, or by arbitration from a third reviewer when necessary. The detailed judgments for each domain across all studies are summarized in Supplementary Table S1. Furthermore, to enhance readability, some secondary figures and tables have been condensed or moved to the Supplementary material.

Data extraction: The required data elements in each study were extracted, including the first author, year of publication, age, number of cases in the trial and control groups, interventions, and outcome indicators.

### 2.6 Statistical analysis

RevMan version 5.4.1 software provided by the Cochrane Collaboration was applied to statistically analyze the extracted outcome indicators. The heterogeneity of clinical outcomes was examined by the Q-test and the I2 statistic test. A fixed-effect model (FEM) was applied when *p* > 0.1 indicated no heterogeneity; a random-effects model (REM) was used when *p* < 0.1 indicated heterogeneity. A predefined strategy was implemented to handle missing data. For missing summary statistics [e.g., means and standard deviations (SDs)], corresponding authors were contacted via email to request complete data. If no response was received within 2 weeks or data were unavailable, a hierarchy of methods was applied. For missing SDs of continuous outcomes assumed to be normally distributed, the missing values were imputed using the average SDs from other studies in the same meta-analysis, calculated from other available statistics (e.g., standard errors, confidence intervals, and *p*-values), or borrowed from a similar study. All imputation processes were thoroughly documented, and their impact on the findings was assessed as part of the sensitivity analysis. Heterogeneity among studies was assessed using the I^2^ statistic. The degree of heterogeneity was interpreted as follows: I^2^ < 25% indicated low heterogeneity, 25% ≤ I^2^ ≤ 50% indicated moderate heterogeneity, and I^2^ > 50% indicated substantial heterogeneity. For the meta-analysis, a fixed-effect model was employed when low heterogeneity was present (I^2^ < 25% and *p* > 0.1 for the Q-test); otherwise, a random-effects model was used. Sensitivity analysis was performed using the leave-one-out method to test the robustness of the pooled results and to identify potential sources of heterogeneity. This was done by sequentially removing each included study and re-running the meta-analysis with the remaining studies. The influence of each study was assessed by observing the changes in the pooled effect size (SMD or RR), its 95% CI, and the I^2^ statistic. A study was considered a notable source of heterogeneity if its exclusion led to a substantial reduction in I^2^ (e.g., a decrease exceeding 50% of the original value), a significant change in the statistical significance of the pooled effect (e.g., *p*-value changing from <0.05 to >0.05, or vice versa), or a reversal in the direction of effect. Such studies were discussed in detail regarding their characteristics. Measurement data were expressed using standardized mean difference (SMD), count data were expressed using relative risk (RR), 95% confidence intervals (CI) indicated the confidence intervals of the parameters, and the results of the tests were presented in forest plots. The RR of the experimental and control groups in the Meta-analysis was used as the horizontal coordinate system, and logRR was used as the vertical coordinate to plot an inverted funnel graph to analyze the distribution pattern of the collected research data to determine whether there was publication bias in the literature.

1. Subgroup and sensitivity analyses.

To further investigate the sources of heterogeneity and test the robustness of the primary results, we pre-specified plans for subgroup and sensitivity analyses.

- Subgroup Analysis: We conducted subgroup analyses based on the following clinically relevant variables when sufficient data were available: (1) the specific type of TCM external therapy (e.g., acupoint application and herbal fumigation/washing); (2) the duration of the treatment course (categorized as ≤4 weeks, >4 weeks to ≤8 weeks, and >8 weeks); and (3) the overall risk of bias (low vs. high/unclear). Heterogeneity within subgroups was assessed using the I^2^ statistic. The differences between subgroups were evaluated using a standard Chi^2^ test, with a *p*-value for subgroup differences of <0.05 considered statistically significant.
- Sensitivity Analysis: A leave-one-out sensitivity analysis was performed to assess the influence of individual studies on the pooled effect estimates. The procedure involved iteratively removing each study and recalculating the summary effect size (MD or OR) and the I^2^ statistic for the remaining studies. We pre-defined that a study would be considered a notable source of heterogeneity or influence if its exclusion led to: (1) a substantial reduction in heterogeneity (specifically, a decrease in the I^2^ value by more than 50% of its original magnitude), (2) a loss of statistical significance for the overall effect (e.g., the *p*-value changing from <0.05 to >0.05), or (3) a marked change (>10%) in the point estimate. The characteristics of such influential studies were then discussed in detail in the results section.

2. Assessment of publication bias.

The potential for publication bias was assessed using a combination of graphical and statistical methods. For outcomes comprising at least 10 studies, Egger’s linear regression test was applied to quantitatively evaluate funnel plot asymmetry. A statistically significant result (*p* < 0.05; p < 0.05) was interpreted as suggestive of potential small-study effects or publication bias. In cases where visual inspection of the funnel plot indicated asymmetry or Egger’s test reached significance, the implications for the robustness of the findings were explicitly discussed in the context of the overall evidence.

### 2.7 Standardization of interventions and data processing

Although all included studies fall under the broad category of “traditional Chinese medicine external therapies”, we recognize significant heterogeneity within this group. To respect and account for this diversity while conducting meta-analyses, we adopted a tiered approach. First, we systematically classified interventions into primary categories based on their physical form and core mechanism of action: Chinese herbal plaster application, Chinese herbal fumigation, Chinese herbal hot compress therapy, and Chinese herbal iontophoresis, among others. Second, we designed and implemented a standardized data extraction process. For each study, we additionally extracted the following critical operational details: core medicinal composition (principal herbs, auxiliary herbs), specific treatment sites (e.g., specific acupoints or muscle groups), duration of a single treatment session, daily or weekly treatment frequency, and total treatment course. For key variables difficult to quantitatively synthesize (e.g., specific intervention categories), we designated them as subgroup analysis variables and conducted sensitivity analyses to assess their impact on the synthesis results.

## 3 Results

### 3.1 Study selection

**Figure 1:**
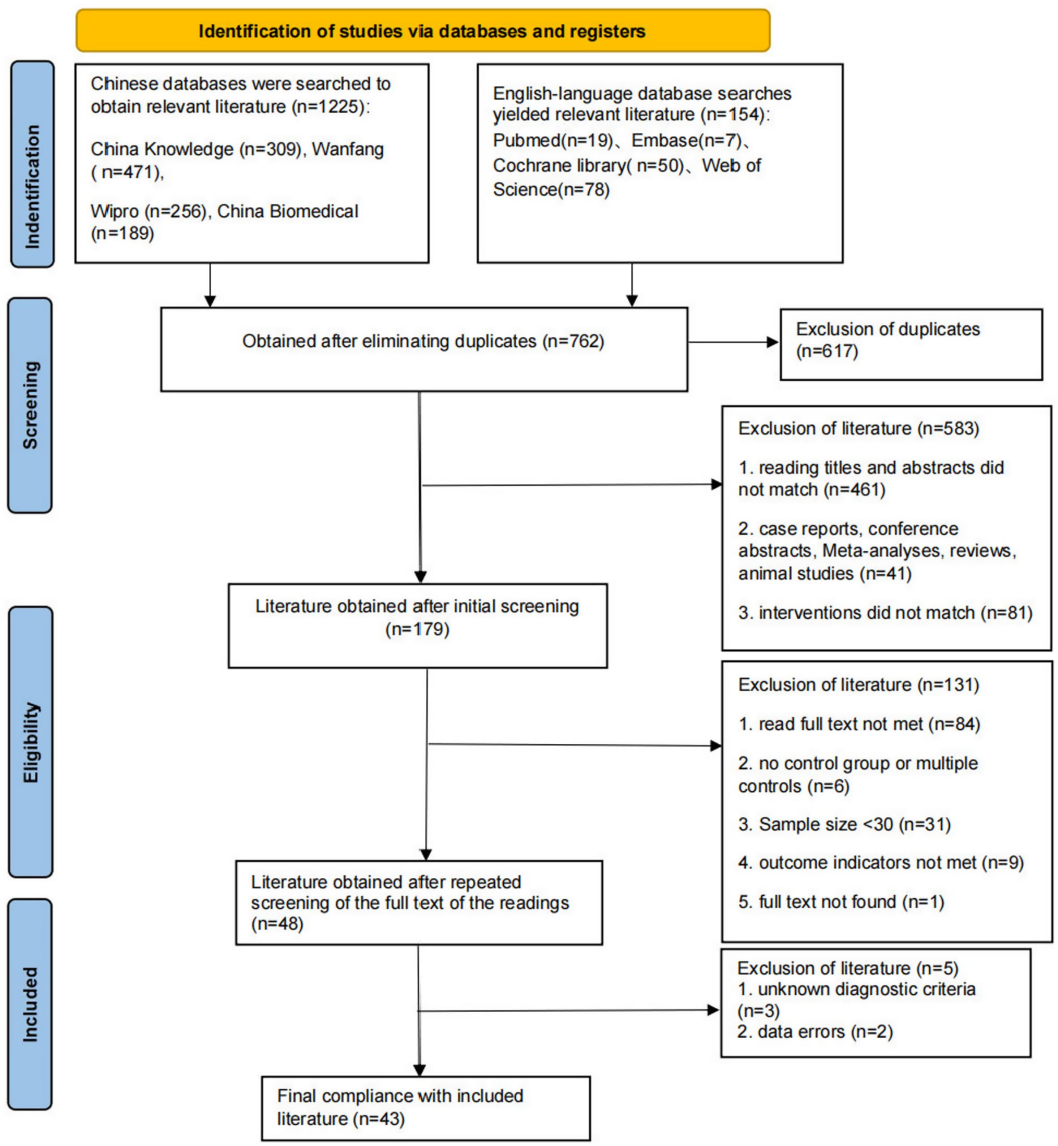
Flowchart of literature search.

The search identified 1,379 records. After deduplication, 617 unique records remained. Title and abstract screening excluded 583 articles. Full-text review of 179 articles, and then the full text of the remaining literature was intensively read, and a total of 131 pieces of literature that did not meet the inclusion criteria were excluded, and 43 pieces of literature were ultimately included (5–47). The screening process and results are shown in Figure 1. A total of 4,186 subjects were included in the 43 papers, and the basic characteristics of the included papers are shown in Table 3. In terms of specific interventions: 19 studies used acupoint application, 10 used TCM fumigation, 9 used TCM washing/fuming, and 2 used TCM intermediate-frequency iontophoresis. In terms of the intervention period, 11 studies chose 4 weeks of intervention, 3 studies chose 6 weeks of intervention, and 6 studies chose 2 weeks of intervention. The results of the included literature showed that there was no significant difference in the index scores of the two groups of patients before treatment (*p* > 0.05); the index scores of the two groups of patients after treatment showed that the therapeutic effect was better than before treatment, and the therapeutic effect of the experimental group was better than the level of the control group, with a significant difference (*p* < 0.05).

**Table 3:** Characteristics of studies included in the meta-analysis.

Author	Year	Sample size	Age (mean ± SD)	Intervention measures				Outcome indicator
		T/C	T/C	T	C	Operational area	days	
Haitao Bai (5)	2009	30/30	55.2 ± 15.4/58.9 ± 10.7	Acupoint application + conventional treatment + rehabilitation	Conventional treatment + rehabilitation	Take Quchi, Jianyu, Waiguan Points for the upper limbs, and apply Huantiao, Yanglingquan and Chengshan points for the lower limbs.	42d	③⑧
Yongli Dong (6)	2011	70/70	63.37 ± 11.45/62.48 ± 10.45	Acupoint application + rehabilitation + medication	Rehabilitation + medication	Quchi, Hegu, Yanglingquan, and Yongquan points	28d	①②
Chunyan Zhao (7)	2012	33/31	53.45 ± 10.14/53.45 ± 10.14	Acupoint application + conventional treatment	Conventional treatment	Jianyu, Naoshu, Binao, Quchi, Shousanli, Waiguan, Hegu, Huantiao, Fengshi, Futu, Xuehai, Yin Yanglingquan, ZuSanli, Sanyinjiao, Yongquan points	30d	①
Xinyu Chen (8)	2013	30/30	63/64	Acupoint application + conventional treatment	Conventional treatment	affected limb	42d	①④
Yu Hu (9)	2014	64/64	40 ~ 74/39 ~ 75	Acupoint application + acupuncture treatment	Acupuncture treatment	Ganshu, Qishu, Qihai, Mingmen Points	40d	①②
Haixia Yang (10)	2015	60/60	63.00 ± 11.22/64.53 ± 10.95	Acupoint application + conventional treatment	Conventional treatment	Waiguan, Hegu, Jianyu, Quchi, Yanglingquan, Zusanli, Xuangzhong, Huantiao points	20d	⑥⑦
Ying Zhou (11)	2015	40/40	72.2 ± 2.5/72.8 ± 2.4	Acupoint application + acupuncture treatment	Acupuncture treatment	Dazhui, QiHai, and Mingmen points	28d	①②
Shiping Sun (12)	2016	150/150	32 ~ 85/36 ~ 81	Acupoint application + conventional treatment	Conventional treatment	Upper limb hemiplegia: Jianyu, Quchi, Waiguan, Hegu, etc. Hemiplegia of the lower limbs: Yanglingquan, Zusanli, Xuangzhong, Huantiao.	14d	①②
Feifei Chong (13)	2018	50/50	61.68 ± 10.28/64.37 ± 9.43	Acupoint application + conventional treatment + acupuncture treatment	Conventional treatment + acupuncture treatment	For the upper limbs, pick up acupoints at Jianyu, Shousanli, and Hegu. Lower limbs: Yinlingquan, Yongquan, and Zusanli.	30d	①②④⑩⑪⑫
Guang Wang (14)	2018	62/62	65.34 ± 3.76/66.34 ± 3.36	Acupoint application + acupuncture treatment	Acupuncture treatment	Gnashu, Danshu, Geshu, Xinshu, Pishu, Shenshu	21d	①②
Minya Zhou (15)	2018	45/45	55.22 ± 6.18/56.89 ± 7.57	Acupoint application + rehabilitation	Rehabilitation	Bilateral Feishu, Pishu, Xinshu, Chizhe, ZuSanli	56d	①②
Mei Wan (16)	2019	40/40	65.45 ± 7.55/64.46 ± 8.26	Acupoint application + rehabilitation	Rehabilitation	For the upper limbs, Jianyu, Binao, Quchi, Shousanli, Waiguan, and Hegu; for the lower limbs, Huantiao, Liangqiu, Zusanli, Fenglong, Sanyinjiao, and Taichong.	84d	③④⑧
Hengkai Lin (17)	2020	41/41	55.63 ± 2.53/56.82 ± 2.64	Acupoint application + acupuncture treatment	Acupuncture treatment	Hegu, ZuSanli, Jianyu, Zusanli, Yongquan, Yinlingquan points	30d	④⑩⑪⑫
Minna Wu (18)	2020	35/35	66.58 ± 11.92/67.86 ± 12.51	Acupoint application + conventional treatment	Conventional treatment	Upper limbs: Hegu, Shousanli, Waiguan, Binao, Quchi, and Jianyu; Lower limbs: Yinlingquan, Yanglingquan, Zusanli, Futu, Xuehai, Sanyinjiao, and Huantiao.	14d	①④
Rui Gong (19)	2021	32/31	64.92 ± 7.13/65.10 ± 6.59	Acupoint application + acupuncture treatment	Acupuncture treatment	Mingmen, Dazhui, Qihai, Jianyu, Shousanli, Hegu, Yinlingquan, Yongquan, Zusanli Points	56d	①③④⑪⑫
Wenjuan Yang(20)	2021	85/85	64.8 ± 5.0/64.5 ± 4.8	Acupoint Application + medication+ rehabilitation	Medication + rehabilitation	Zusanli, Yongquan, Yanglingquan, Hegu, Shousanli, and Jianyu on the affected side	60d	①②③⑨
Tianxiao Lan (21)	2023	43/43	60.69 ± 3.29/60.65 ± 3.24	Acupoint application + acupuncture treatment	Acupuncture treatment	Jianjing, Yongquan, Quchi Points	30d	①③④⑩⑪⑫
Ping Liu (22)	2023	43/43	63.02 ± 5.51/62.46 ± 5.43	Acupoint application + acupuncture treatment	Acupuncture treatment	Xinshu, Pishu, Ganshu, Shenshu	84d	①②③④⑥
Lina Sun (23)	2023	74/74	64.41 ± 9.47/64.37 ± 9.50	Acupoint application + medication + rehabilitation	Medication + rehabilitation	Jianyu, Quchi, Hegu, and Waiguan, Zusanli, Huantiao, Liangqiu, Yongquan, and Yanglingquan points.	42d	①②③④
Jianxiao Huang (24)	2011	45/45	62.0 ± 4.92/59.6 + 5.38	Herbal fumigation + rehabilitation	Rehabilitation	Affected limb	28d	④⑧
Huali Liu (25)	2011	41/39	39 ~ 82/39 ~ 82	Herbal fumigation + rehabilitation	Rehabilitation	-	29d	①②
Shihui Wu (26)	2012	41/37	49 ~ 75/49 ~ 75	Herbal fumigation + medication+ acupuncture treatment	Medication + acupuncture treatment	Affected limb	20d	①
Xuan Liu (27)	2016	48/48	44.17 ± 9.23/47.14 ± 12.02	Herbal fumigation + conventional treatment + rehabilitation	Conventional treatment + rehabilitation	Affected limb	14d	①②③④
Kai Cai (28)	2017	63/63	49.35 ± 6.01/48.62 ± 5.83	Herbal fumigation + rehabilitation	Rehabilitation	Above the ankle to the knee	14d	④
Guohui Liao (29)	2017	44/36	45 ~ 70/45 ~ 70	Herbal fumigation + conventional treatment	Conventional treatment	affected limb	30d	①②③⑤
Caiyun Chu (30)	2018	61/61	54.08 ± 1.99/54.15 ± 2.03	Herbal fumigation + acupuncture treatment	Acupuncture treatment	The affected side of Shousanli, Waiguan, Houxi, Yinbao, Sanyinjiao, Yinlingquan, and other points	21d	①②③
Lihong Jiang (31)	2018	40/40	58.07 ± 9.35/58.26 ± 9.63	Herbal external cleansing + conventional treatment	Conventional treatment	Affected limb	14d	①②③⑥
Lijuan Li (32)	2019	40/40	63.47 ± 3.48/64.76 ± 3.86	Herbal fumigation + medication+ acupuncture treatment	Medication + acupuncture treatment	Em (typography)	15d	④⑧
Jingyi Ke(33)	2021	30/30	62.2 ± 5.5/62.5 ± 5.7	Herbal fumigation + rehabilitation	Rehabilitation	Affected limb	28d	①③
Xiansong Liu (34)	2021	50/50	66.12 ± 10.47/65.24 ± 10.53	Herbal fumigation + acupuncture treatment	Acupuncture treatment	Affected limb	60d	①③⑤⑦⑧
Ying Zhang (35)	2007	30/30	65.4 ± 8.71/66.58 ± 10.17	Herbal fumigation + rehabilitation	Rehabilitation	Em (typography)	28d	①③
Zhixing Wang (36)	2013	78/78	65.12 ± 4.17/63.88 ± 4.13	Herbal fumigation + acupuncture treatment	Acupuncture treatment	Lower-leg knee	60d	①
Dongdi Zhao (37)	2015	50/50	59.35 ± 7.06/60.95 ± 7.67	Herbal fumigation + acupuncture treatment + rehabilitation	Acupuncture treatment +rehabilitation	Affected limb	30d	①⑦
Jieming Wang (38)	2017	40/40	56 ~ 67/56 ~ 67	Herbal fumigation + rehabilitation + massage therapy	rehabilitation+ massage therapy	em (typography)	90d	①⑧
Yan Yang (39)	2020	34/34	62.24 ± 6.29/62.09 ± 9.82	herbal fumigation + conventional treatment	conventional treatment	em (typography)	28d	①②③⑥
Qiuyun Ma(40)	2021	41/41	60 ~ 80/60 ~ 80	Herbal fumigation + rehabilitation + conventional treatment	Rehabilitation + conventional treatment	Directing Vessel, Bladder Meridian	28d	①⑥⑨
Yuxia Sheng (41)	2021	40/40	56.3 ± 2.1/56.2 ± 2.2	herbal fumigation + rehabilitation	Rehabilitation	affected limb	14d	①
Tianye Yang (42)	2022	46/46	62.45 ± 5.25/62.38 ± 5.36	herbal fumigation + rehabilitation + Acupuncture treatment	Rehabilitation + Acupuncture treatment	affected limb	28d	③④
Hui Yang (43)	2023	55/54	57.32 ± 7.14/58.76 ± 8.37	herbal fumigation + rehabilitation	Rehabilitation	Em (typography)	60d	③⑥⑦⑧⑨
Zhuangmiao Li (44)	2019	43/43	40 ~ 80/40 ~ 80	Chinese herbal ironing + conventional treatment + rehabilitation	Conventional treatment + rehabilitation	Directing Vessel, Bladder Meridian	28d	③
Ziqin Wang (45)	2021	35/35	56.87 ± 5.23/55.34 ± 5.17	Chinese herbal ironing + Acupoint Application + conventional treatment	conventional treatment	affected limb	84d	③
Hui Zhang (46)	2016	30/30	58 ± 12/57 ± 11	Chinese Medicine Ion Importation + conventional treatment	conventional treatment	The meridian nodes at the hip, knee, ankle, and foot through which the foot syncopal meridian tendons, foot taiyin meridian tendons, foot solar meridian tendons, and foot shaoyang meridian tendons circulate.	28d	④
Mengting Wu (47)	2017	50/50	-	Chinese Medicine Ion Importation + medication + rehabilitation	Medication + rehabilitation	For upper limb points, take Quchi and Waiguan; for lower limb points, take Zusanli and Sanyinjiao.	28d	③⑤

A total of 4,186 patients were included. T: Test group; C: Control group; −: Not mentioned; ① Total effective rate, ② Cure rate, ③ FMA score, ④ Barthel index, ⑤ Modified Barthel index, ⑥ NIHSS score, ⑦ ADL score, ⑧ MAS score, ⑨ BBS score, ⑩ MMT score, ⑪ PAI-1 level, ⑫ t-PA level.

### 3.2 Quality assessment of the included literature

**Figure 2:**
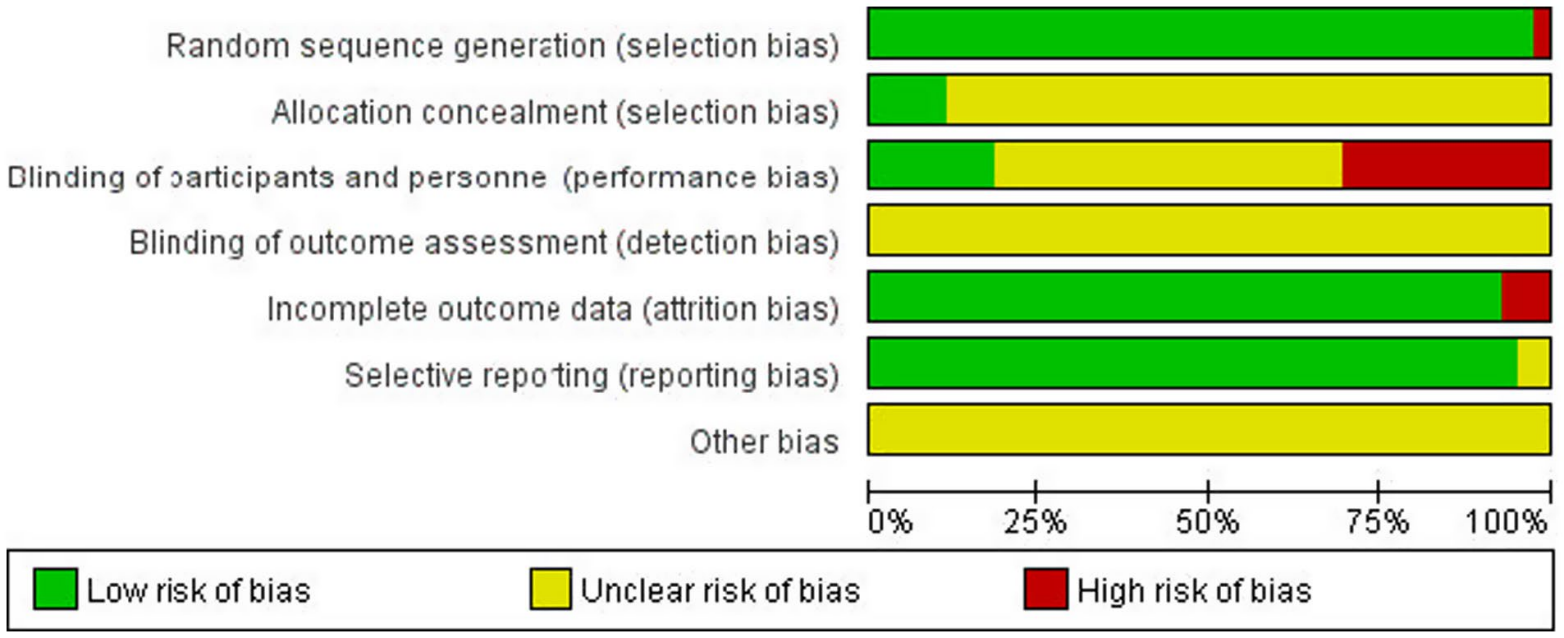
Risk of bias assessment chart.

**Figure 3:**
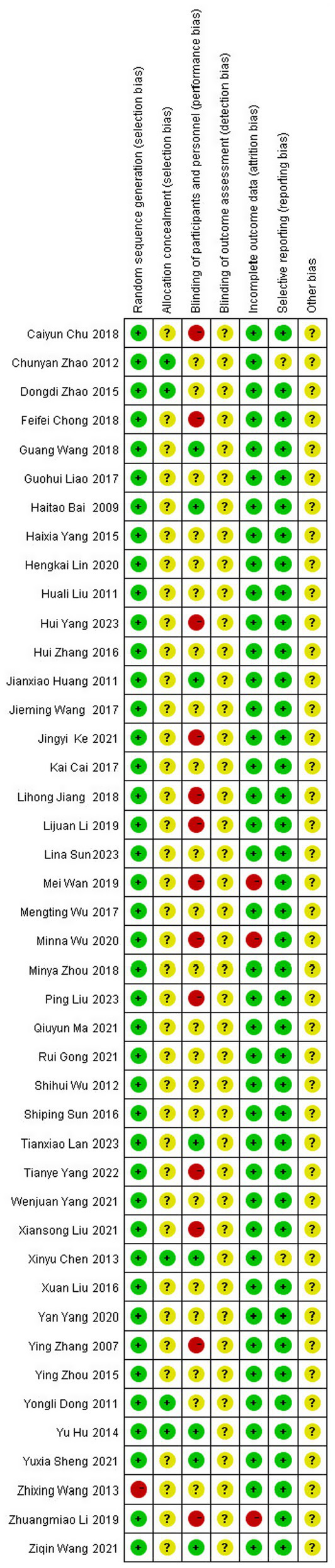
Summary of the risk of bias of the included literature.

Literature risk of bias evaluation was performed according to the Cochrane Handbook for Systematic Evaluation 6.2, and the results are shown in Figures 2, 3.

### 3.3 Meta-analysis results

#### 3.3.1 Meta-analysis of overall effectiveness rate

**Figure 4:**
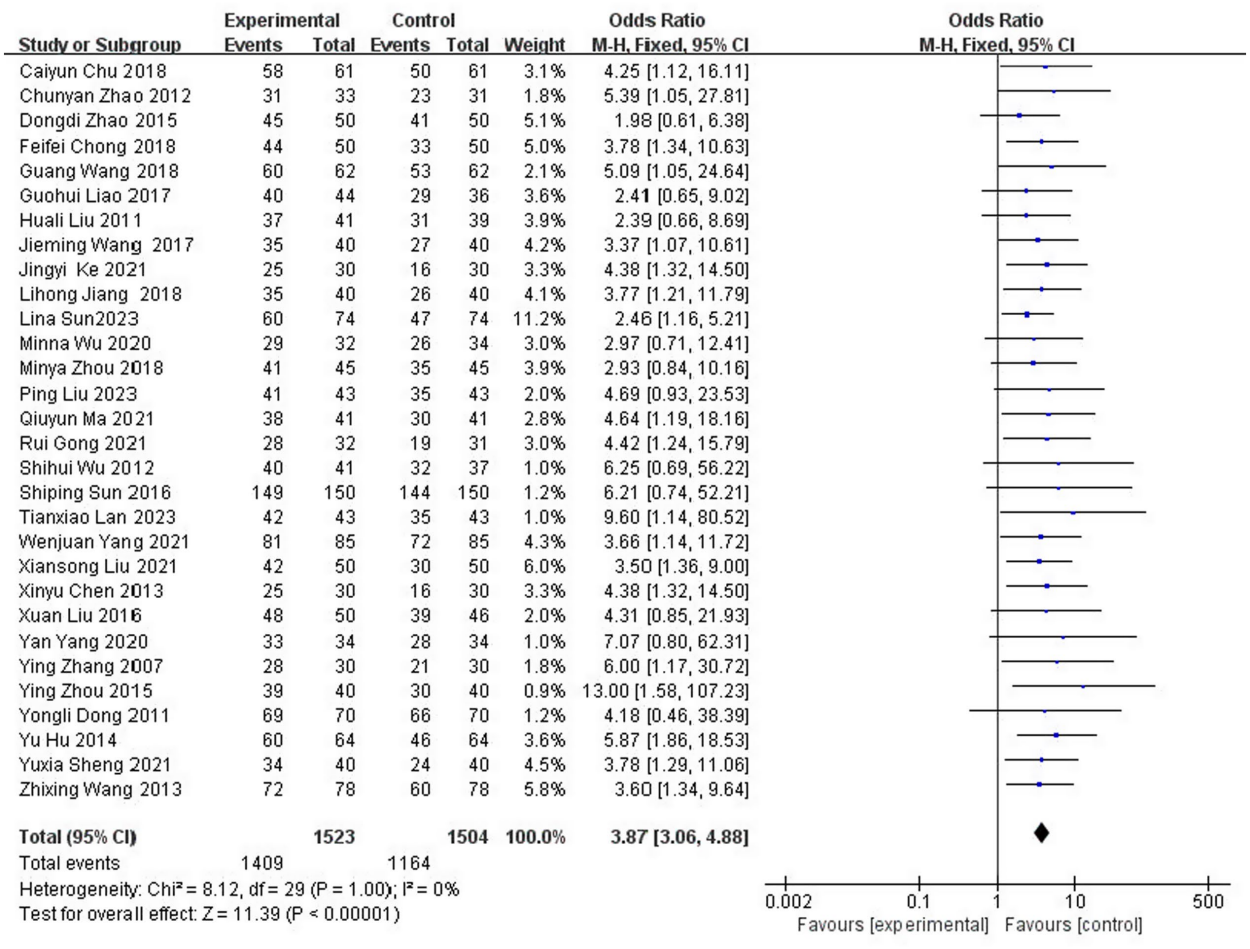
Forest plot of the total effective rate.

A total of 30 studies (6–9, 11–15, 18–23, 25–27, 29–31, 33–41) were included to report the total clinical effective rate after treatment, involving 3,027 patients (accounting for 72.3% of the total 4,186 patients included in this meta-analysis), of which 1,523 patients in the experimental group were effective in 1,409 cases, and 1,504 patients in the control group were effective in 1,164 cases. The 3,027 patients included in this specific analysis represent a subset of the total 4,186 patients from the included studies. The inclusion criteria for this outcome were based on studies that explicitly reported dichotomous data for treatment effectiveness (cured, markedly effective, effective, and ineffective), allowing for the calculation of the total effective rate. Heterogeneity was low (I^2^ = 0%, *p* = 1.00), so FEM was used. TCM external application significantly increased the total effective rate [OR = 3.87, 95% CI: (3.06, 4.88), *p* < 0.00001] (Figure 4).

#### 3.3.2 Meta-analysis of cure rate

**Figure 5:**
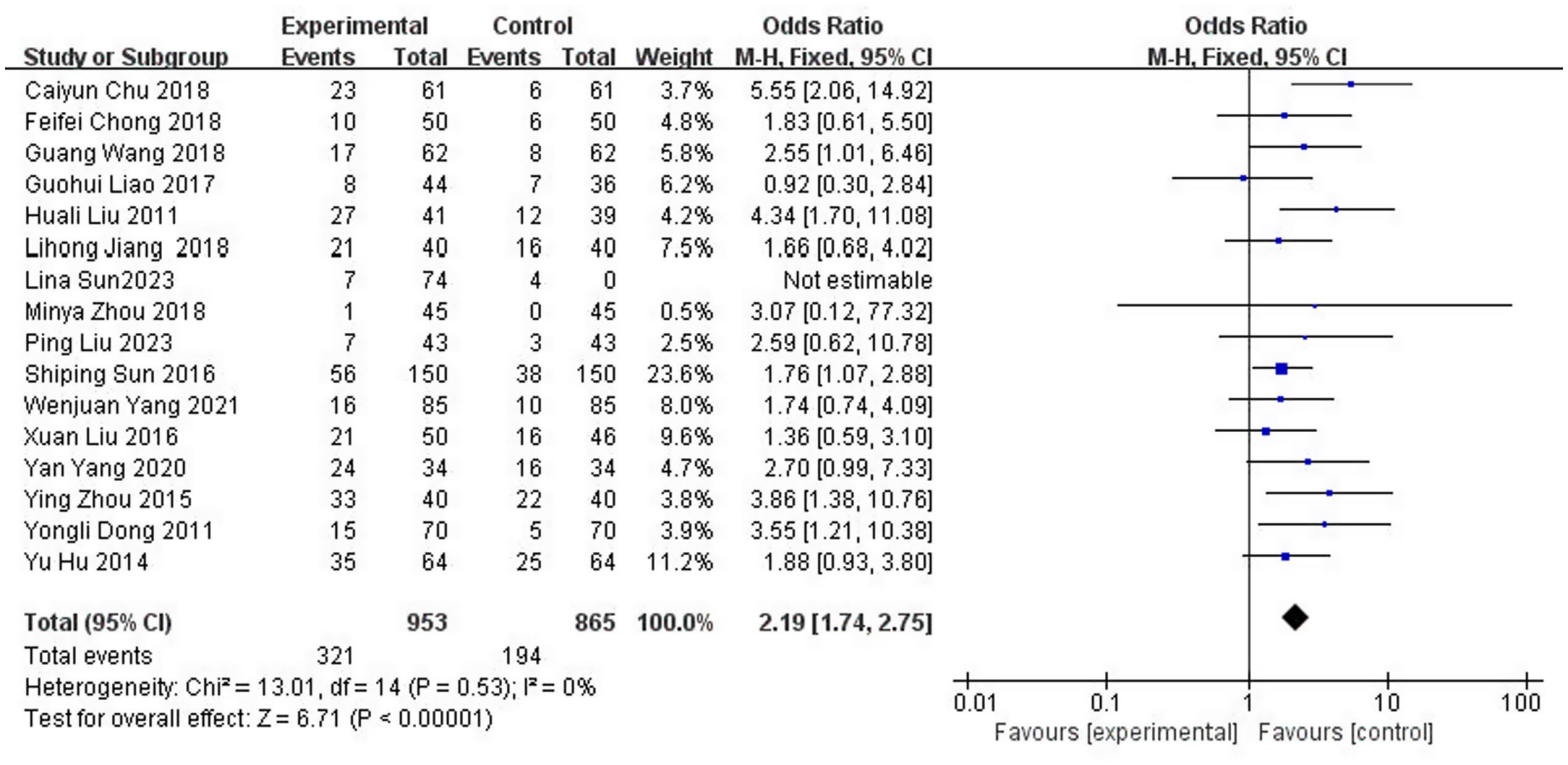
Forest plot of cure rate.

A total of 16 studies (6, 9, 11–15, 20, 22, 23, 25, 27, 29–31, 39) reported the clinical cure rate after treatment, involving 1,818 patients (43.4% of the total 4,186 patients), of which 953 patients in the experimental group were cured in 321 cases and 865 patients in the control group were cured in 194 cases. The results of the heterogeneity test (I^2^ = 0%, *p* = 0.53) suggested homogeneity among the selected literature for this study, and FEM was chosen for meta-analysis. The cure rate was significantly higher in the test group [OR = 2.19, 95% CI: (1.74, 2.75)] (Figure 5).

#### 3.3.3 Meta-analysis of FMA scores

**Figure 6:**
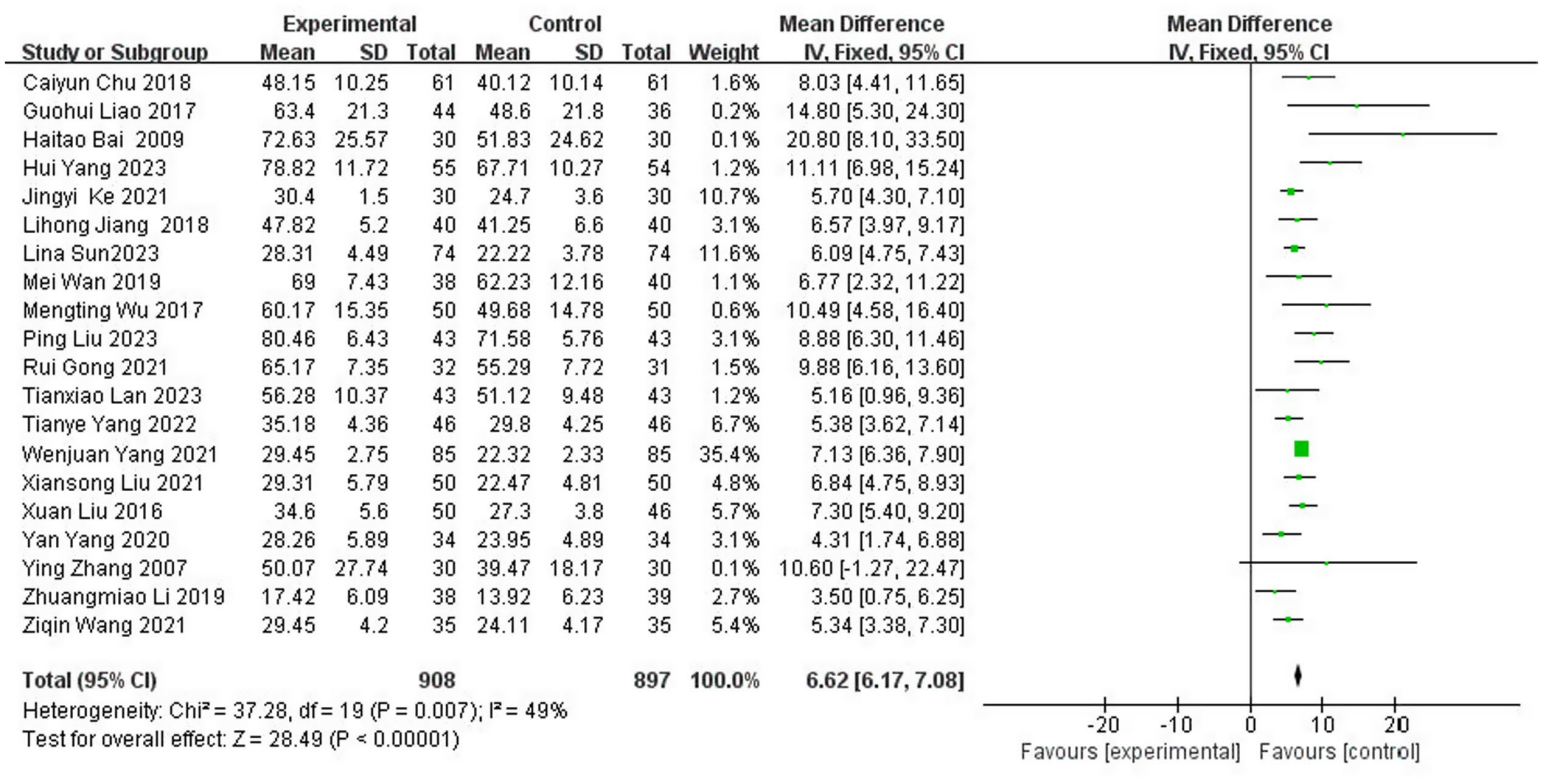
Forest plot of FMA scores.

A total of 20 studies (5, 16, 19–23, 27, 29–31, 33–35, 39, 42–45, 47) reported the Fugl-Meyer Motor Function Score (FMA) scores after treatment, which involved 1,805 patients (representing 43.1% of the total 4,186 patients), of which 908 patients were in the experimental group and 897 patients were in the control group. The results of the heterogeneity test (I^2^ = 49%, *p* = 0.01) indicated moderate heterogeneity among the included studies; consequently, FEM was chosen for meta-analysis. The results showed that compared with the control group, the application of herbal topical medicine significantly improved the patients’ Fugl-Meyer motor function score (FMA) [MD = 6.62, 95% CI: (6.17, 7.08), *p* < 0.00001] (Figure 6).

#### 3.3.4 Meta-analysis of Barthel Index score, modified Barthel Index score, and ADL score

##### 3.3.4.1 Barthel Index score

**Figure 7:**
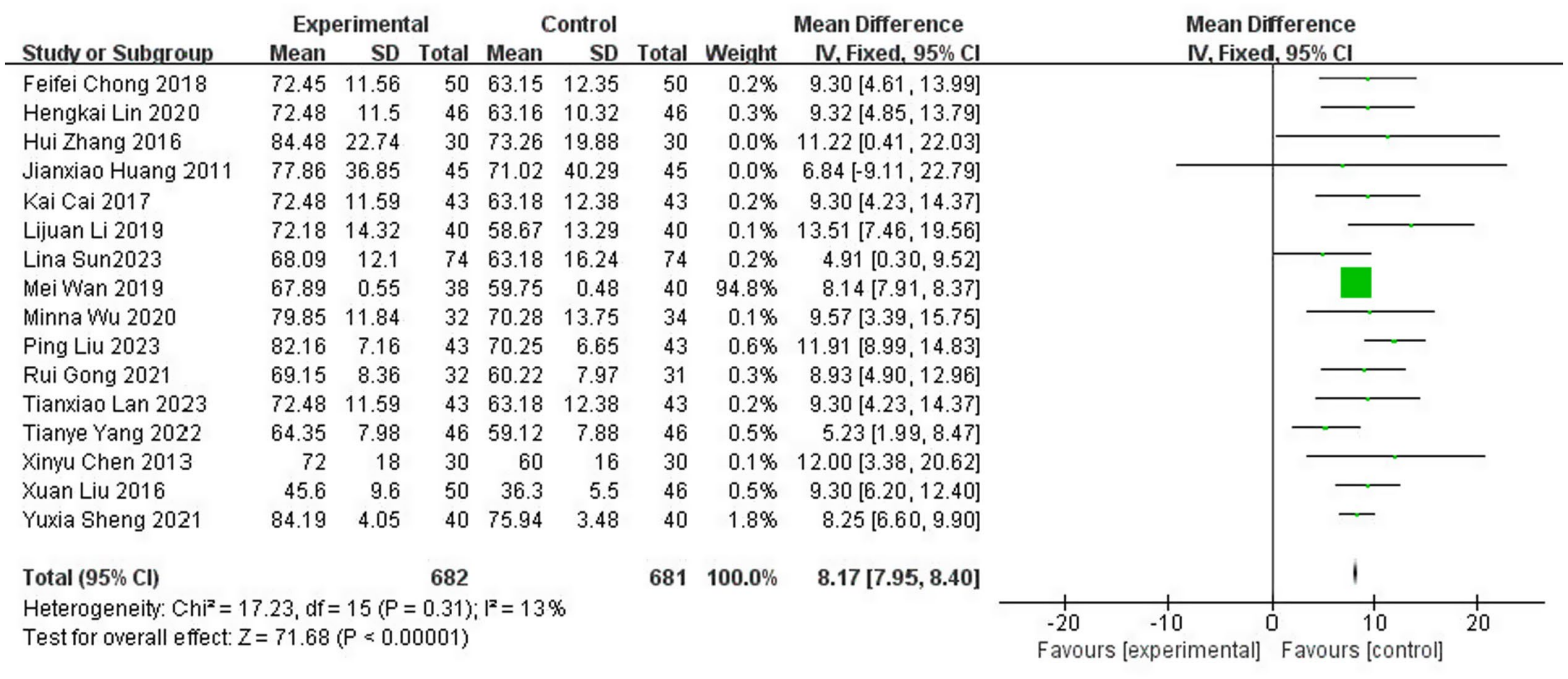
Forest plot of the Barthel Index score.

A total of 16 studies (8, 13, 16–19, 21–24, 27, 28, 32, 41, 42, 46) reported on the ability to perform daily living assessment (Barthel Index Score), with a total of 1,363 patients (32.6% of the total 4,186 patients), of which 682 were in the test group and 681 in the control group. The results of the heterogeneity test (I2 = 13%, *p* = 0.31) suggested homogeneity among the selected literature for this study, and FEM was chosen for Meta-analysis. The results showed that compared with the control group, the application of topical Chinese medicine significantly improved the patients’ Barthel Index score [MD = 8.17, 95% CI: (7.95, 8.40), *p* < 0.00001] (Figure 7).

##### 3.3.4.2 Modified Barthel Index score

**Figure 8:**
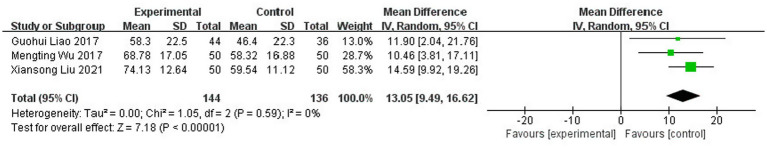
Forest plot of Modified Barthel Index score.

A total of three studies (29, 34, 47) reported on the ability to perform daily living assessment (Modified Barthel Index Score), with a total of 280 patients, including 144 in the test group and 136 in the control group. The results of the heterogeneity test (I^2^ = 0%, *p* = 0.59) suggested homogeneity among the selected literature for this study, and FEM was chosen for meta-analysis. The results showed that the application of topical herbal medicine significantly improved the MBI scores of patients compared with the control group [MD = 13.05, 95% CI: (9.49, 16.62), *p* < 0.00001] (Figure 8).

##### 3.3.4.3 ADL score

**Figure 9:**
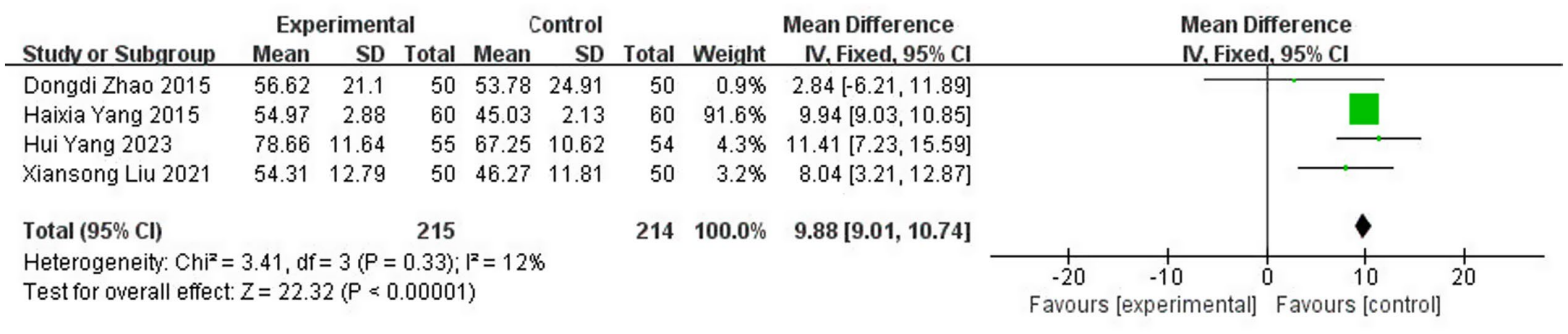
Forest plot of ADL score.

A total of four studies (10, 34, 37, 43) reported ADL scores with 429 patients, including 215 in the experimental group and 214 in the control group. The results of the heterogeneity test (I^2^ = 12%, *p* = 0.33) suggested homogeneity among the selected literature for this study, and FEM was chosen for meta-analysis. The results showed that the application of topical herbal medicine significantly improved patients’ ADL scores compared with the control group [MD = 9.88, 95% CI: (9.01, 10.74), *p* < 0.00001] (Figure 9).

#### 3.3.5 Meta-analysis of NIHSS score

**Figure 10:**
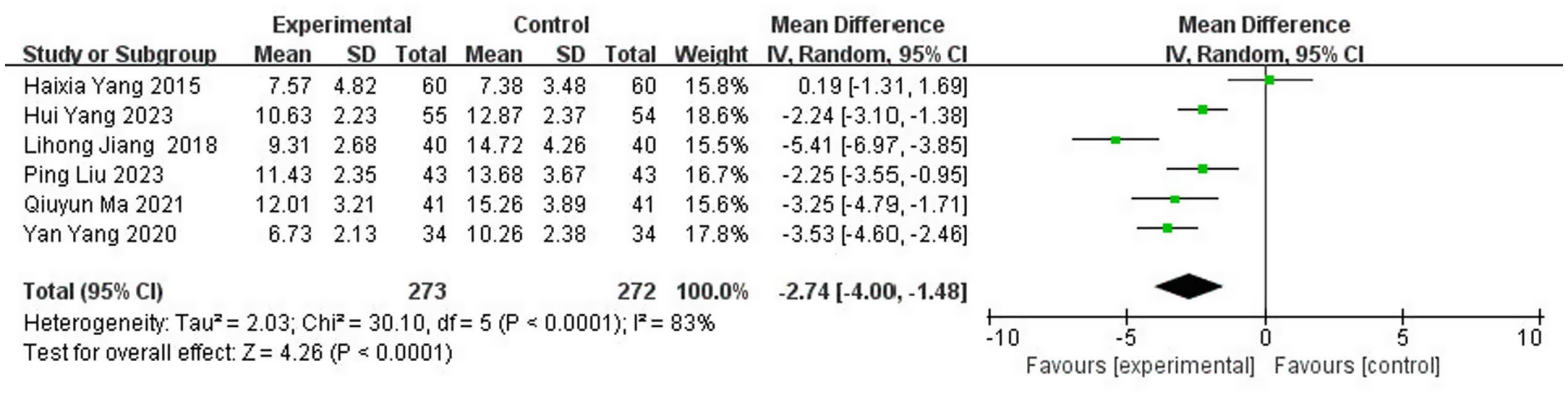
Forest plot of NIHSS score.

A total of six studies (10, 22, 31, 39, 40, 43) reported the NIHSS scores after treatment, involving 545 patients (13.0% of the total 4,186 patients), including 273 patients in the test group and 272 patients in the control group. The initial overall heterogeneity test indicated substantial heterogeneity (I^2^ = 83%, p < 0.00001), necessitating the use of a random effects model (REM) for the primary meta-analysis (Figure 10).

##### 3.3.5.1 Subgroup analysis by treatment duration: elucidating the source of heterogeneity

**Figure 11:**
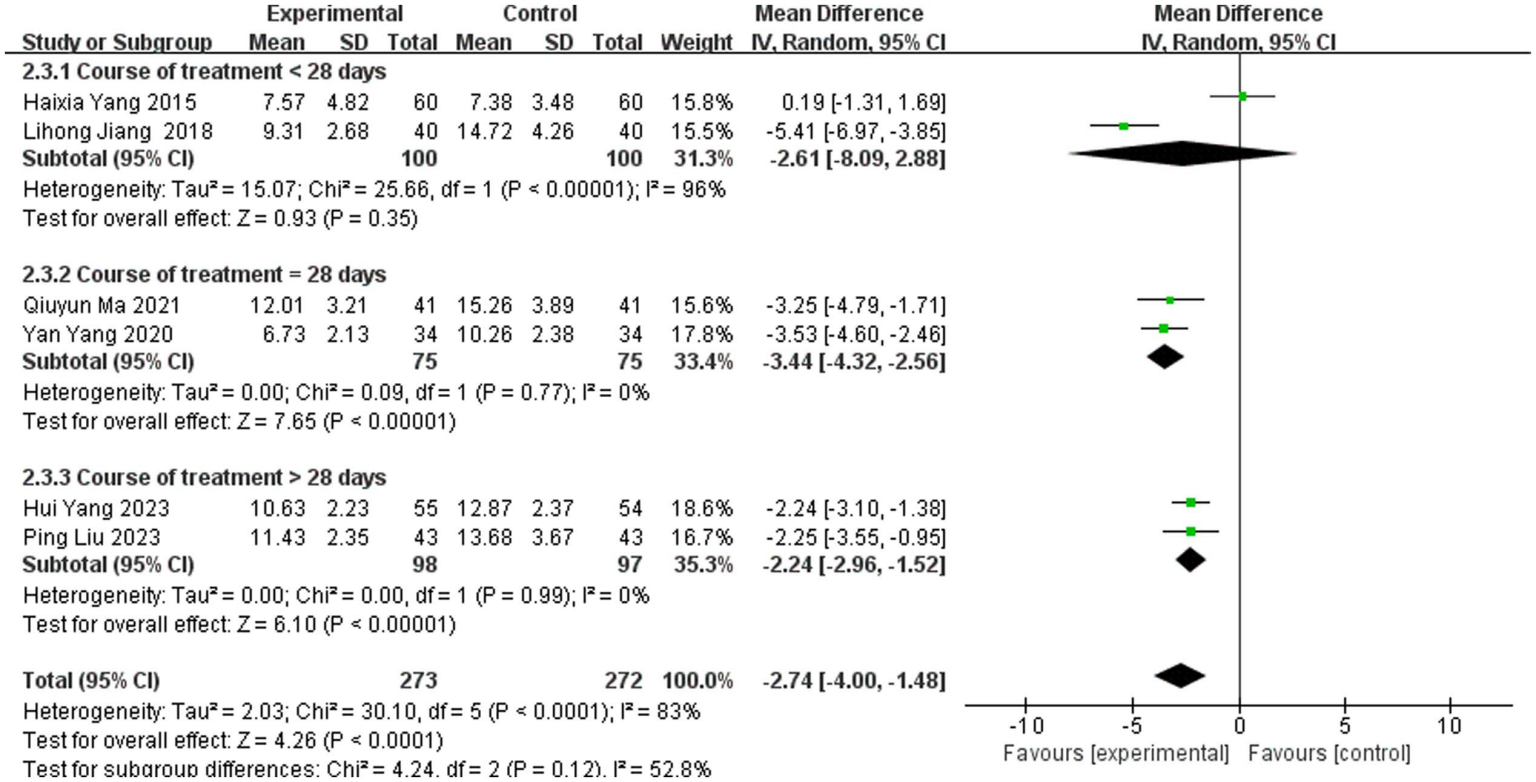
Forest plot of NIHSS score subgrouped by treatment duration.

To investigate the substantial heterogeneity observed in the overall analysis, we conducted an *a priori* subgroup analysis based on treatment duration—a clinically salient variable hypothesized to influence the extent of neurological recovery. Studies were stratified into three predefined categories: short term (<28 days), standard term (=28 days), and extended term (>28 days) therapy (Figure 11).

The results were striking and revealed a clear pattern:

In the short-term group [<28 days, 2 studies (10, 31)], the pooled effect was MD = −2.61 [95% CI: −8.12, 2.90], but this estimate was highly unreliable due to extreme heterogeneity (I^2^ = 95%, *p* < 0.00001). The confidence interval is exceptionally wide, crossing the null value (MD = 0), indicating profound inconsistency and a lack of statistical power to draw a meaningful conclusion for this subgroup.

In contrast, both the standard-term group (=28 days, 2 studies (39, 40); MD = −3.44 [95% CI: −4.32, −2.55]) and the extended-term group (>28 days, 2 studies (22, 43); MD = −2.24 [95% CI: −2.96, −1.52]) demonstrated statistically significant benefits and, crucially, perfect homogeneity (I^2^ = 0% for both).

*Interpretation of subgroup findings*: This subgroup analysis provides a compelling explanation for the overall heterogeneity. The extreme heterogeneity appears to be driven almost exclusively by the two short-duration studies. The perfect consistency (I^2^ = 0%) observed in the 28-day and longer-duration groups suggests that treatment duration is a key effect modifier. The biological plausibility for this is strong: stroke recovery, particularly neuroplasticity and the reorganization of neural circuits, is a time-dependent process. A treatment duration of less than 28 days may be insufficient to produce a stable and measurable effect on the NIHSS, leading to variable and unpredictable outcomes. The convergence of effects in the ≥28-day groups indicates that this may represent a critical threshold for achieving a consistent, reproducible therapeutic response in neurological function, as measured by the NIHSS.

##### 3.3.5.2 Sensitivity analysis: testing the robustness of the conclusion

Guided by the results of the subgroup analysis, we performed a sensitivity analysis to test the robustness of the overall finding. We systematically excluded the two studies identified as the primary sources of heterogeneity Yang et al. (10) and Jiang (31) both of which featured the shortest treatment courses (<28 days).

**Figure 12:**
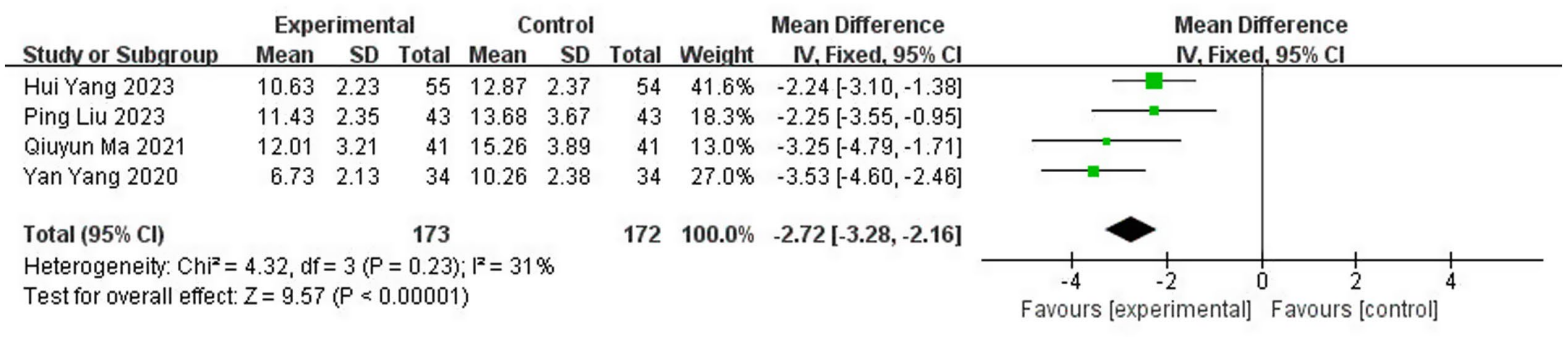
Sensitivity analysis of NIHSS score.

Upon their exclusion, the heterogeneity among the remaining four studies dropped substantially from I^2^ = 83% to I^2^ = 31% (*p* = 0.23), confirming that these two studies were the major contributors to the initial statistical inconsistency. With low residual heterogeneity, a fixed effect model (FEM) was appropriate. This more robust analysis yielded a precise and statistically significant result: the application of traditional Chinese medicine topical method significantly reduced neurological deficit scores [MD = −2.72, 95% CI: (−3.28, −2.16), *p* < 0.00001] (Figure 12).

*Interpretation of sensitivity analysis*: This sensitivity analysis serves two critical purposes:

It validates the subgroup finding: The dramatic reduction in heterogeneity upon removing the short-duration studies confirms that treatment duration was a central cause of the initial statistical heterogeneity.

It provides a more reliable effect estimate: The analysis based on the homogeneous subset of studies (I^2^ = 31%) provides a more confident and precise estimate of the true treatment effect. The fact that the result remains highly significant (*p* < 0.00001) with a narrow confidence interval after removing the outliers strengthens the conclusion that TCM external therapy is efficacious, and the initial overall estimate was not merely an artifact of heterogeneous studies.

In summary, the combination of subgroup and sensitivity analyses moves beyond simply acknowledging heterogeneity to successfully diagnosing its source and demonstrating that the core conclusion of a beneficial treatment effect is robust and dependable when considering studies with an adequate treatment duration.

#### 3.3.6 Meta-analysis of MAS score

**Figure 13:**
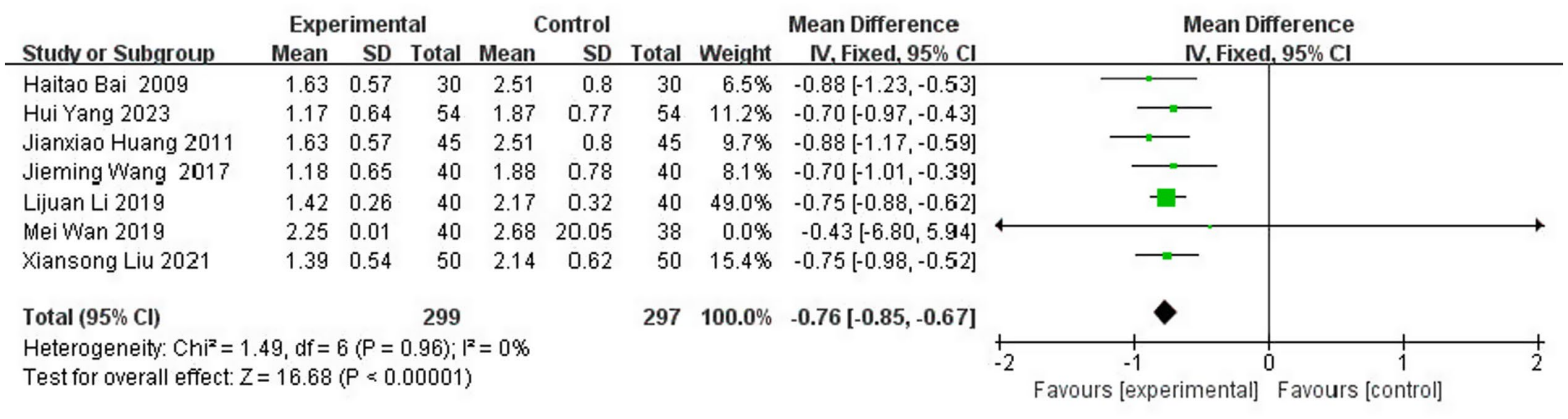
Forest plot of MAS score.

A total of seven studies (5, 16, 24, 32, 34, 38, 43) reported the MAS scores after treatment, involving 596 patients (14.2% of the total 4,186 patients), including 299 patients in the experimental group and 297 patients in the control group. The results of the heterogeneity test (I^2^ = 0%, *p* = 0.96) suggested homogeneity among the literature selected for this study, and FEM was chosen for meta-analysis. The results showed that compared with the control group, the application of topical Chinese medicine could significantly reduce the degree of neurological deficits in patients [MD = -0.76, 95% CI: (−0.85, −0.67), *p* < 0.00001] (Figure 13).

#### 3.3.7 Meta-analysis of BBS score

**Figure 14:**
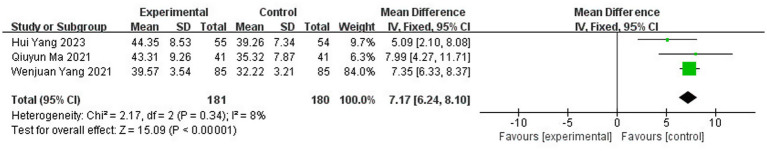
Forest plot of BBS score.

A total of three studies (20, 40, 43) reported the BBS scores after treatment, involving 361 patients (8.6% of the total 4,186 patients), including 181 patients in the test group and 180 patients in the control group. The results of the heterogeneity test (I^2^ = 8%, *p* = 0.34) suggested homogeneity among the selected literature in this study, and FEM was chosen for meta-analysis. The results showed that compared with the control group, the application of topical herbal medicine significantly improved the balance function of patients [MD = 7.17, 95% CI: (6.24, 8.10), *p* < 0.00001] (Figure 14).

#### 3.3.8 Meta-analysis of MMT score

**Figure 15:**
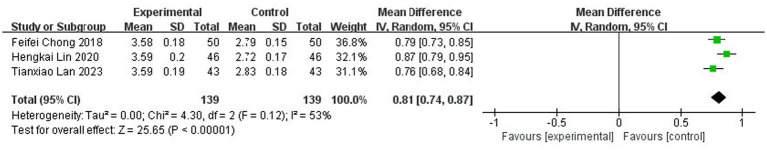
Forest plot of MMT score.

A total of three studies (13, 17, 21) reported treated MMT scores involving 278 patients (6.6% of the total 4,186 patients), including 139 patients in the test group and 139 patients in the control group. The overall heterogeneity test showed significant heterogeneity (I^2^ = 53%, *p* = 0.12), prompting the use of a random effects model (REM) for the primary meta-analysis. The initial results indicated that compared with the control group, the application of herbal topical medicine could significantly improve patients’ MMT scores [MD = 0.81, 95% CI: (0.74, 0.87), *p* < 0.00001] (Figure 15).

Sensitivity Analysis and In-depth Interpretation of Heterogeneity Sensitivity analysis was conducted by excluding the three papers one by one to interrogate the robustness of the pooled estimate and to understand the nature of the observed heterogeneity. This procedure functioned as a diagnostic tool, revealing that the study by Lin (17) was the primary contributor to the statistical heterogeneity. Its exclusion resulted in a definitive resolution of heterogeneity, with the I^2^ statistic dropping from 53 to 0% (*p* = 0.56).

The interpretation of this finding is two-fold. First, from a statistical perspective, the transition from substantial heterogeneity (I^2^ = 53%) to perfect homogeneity (I^2^ = 0%) confirms that the variability was not random but was attributable to the distinct characteristics of a single study. This allows for a more confident interpretation of the pooled result from the two consistent studies. Second, from a clinical and methodological perspective, identifying the outlier study enables a focused inquiry into its unique attributes. The notably younger patient population in Lin (17) provides a biologically plausible explanation for the observed discrepancy. Younger age is a well-established positive prognostic factor in stroke recovery, likely facilitating a different, potentially enhanced, response to therapy compared to older cohorts in the other trials. This does not invalidate the findings of Lin (17) but rather contextualizes them, suggesting that the magnitude of the treatment effect on MMT may be influenced by patient age.

**Figure 16:**
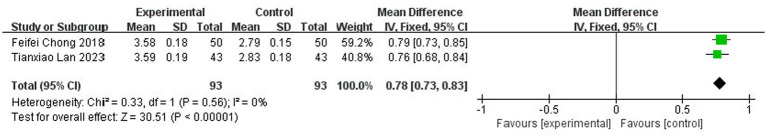
Sensitivity analysis of MMT score.

A fixed effect model (FEM) was then applied to the two homogeneous studies, yielding a result that remained highly statistically significant [MD = 0.78, 95% CI (0.73, 0.83), *p* < 0.00001]. (Figure 16). Crucially, the point estimate of 0.78 is very close to the initial estimate of 0.81, demonstrating that while the heterogeneity was real and traceable, it did not fundamentally alter the primary conclusion regarding the efficacy of the intervention. This strengthens the overall finding by showing that a consistent, significant treatment effect exists across a more homogeneous subset of the evidence.

#### 3.3.9 Meta-analysis of serum levels of fast inhibitor enzyme-1 (PAI-1) and tissue-type plasminogen activator (t-PA)

##### 3.3.9.1 PAI-1

**Figure 17:**
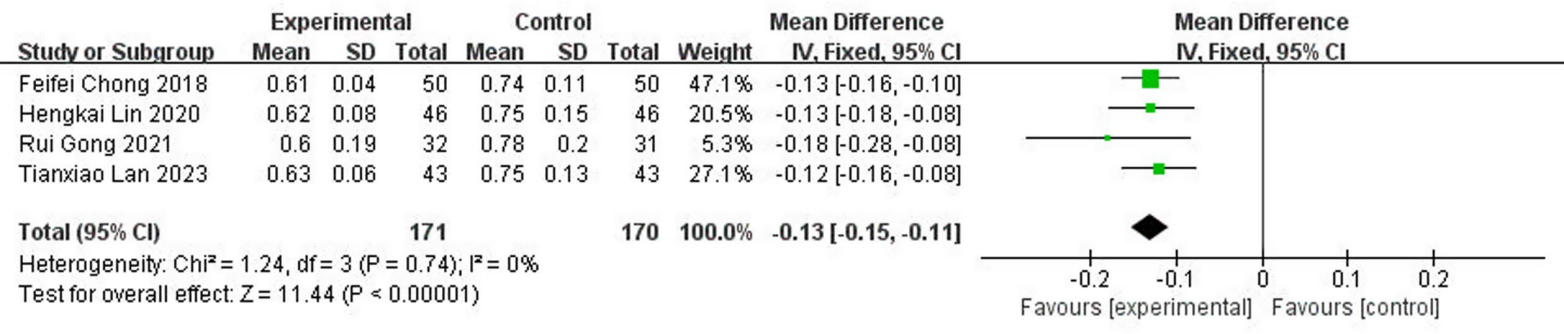
Forest plot of PAI-1.

A total of four studies (13, 17, 19, 21) reported serum levels of fast inhibitory enzyme-1 after treatment, involving 341 patients (8.1% of the total 4,186 patients), of which 171 patients were in the test group and 170 were cured. The results of the heterogeneity test (I^2^ = 0%, *p* = 0.74) suggested homogeneity among the literature selected for this study, and FEM was chosen for meta-analysis. The results showed that there was a statistically significant difference between the serum levels of fast inhibitor enzyme-1 in the test group and the control group [MD = −0.13, 95% CI: (−0.15, −0.11), *p* < 0.00001] (Figure 17).

##### 3.3.9.2 t-PA

**Figure 18:**
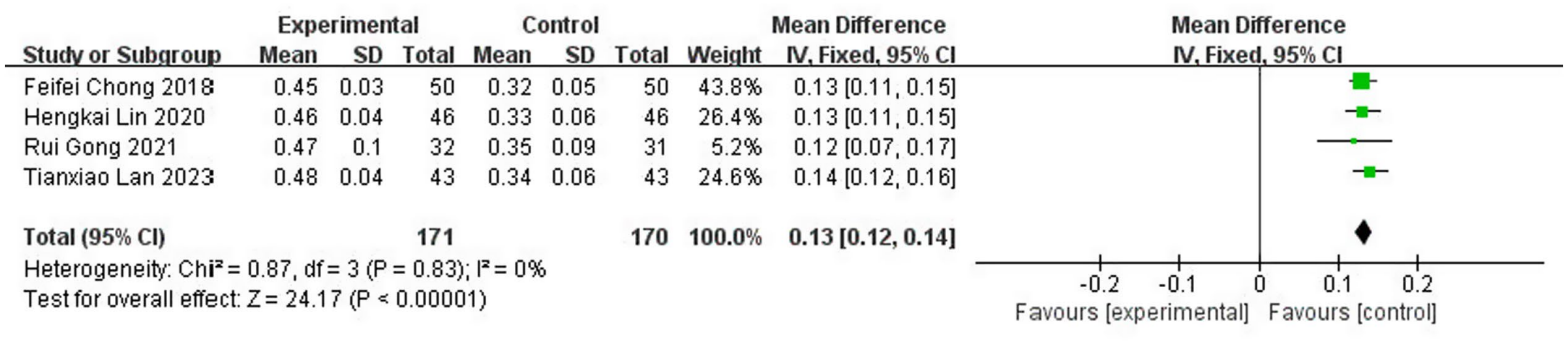
Forest plot of t-PA.

A total of four studies (13, 17, 19, 21) reported tissue-type fibrinogen activator (t-PA) levels after treatment, involving 341 patients (8.1% of the total 4,186 patients), including 171 patients in the test group and 170 cured patients. The results of the heterogeneity test (I^2^ = 0%, *p* = 0.83) suggested homogeneity among the literature selected for this study, and FEM was chosen for meta-analysis. The results showed that there was a statistically significant difference between the levels of tissue-type fibrinogen activator (t-PA) in the test group and the control group [MD = 0.13, 95% CI: (0.12, 0.14), *p* < 0.00001] (Figure 18).

### 3.4 Publication bias analysis

The potential for publication bias was rigorously assessed through a combination of visual and statistical methods.

**Figure 19:**
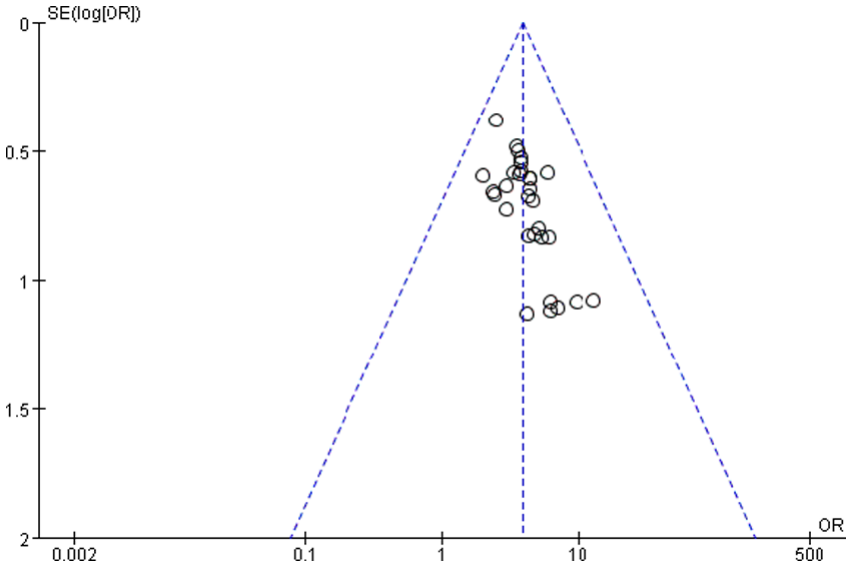
Funnel plot of the total efficiency.

Visual inspection of the funnel plot (Figure 19) revealed pronounced asymmetry, with a notable absence of small-scale studies exhibiting null or negative findings in the lower-left quadrant. This pattern is suggestive of a potential bias against the publication of smaller, non-significant studies.

This visual impression was quantitatively confirmed by two complementary statistical tests. Egger’s linear regression test, which assesses funnel plot asymmetry, yielded a statistically significant intercept (intercept = 1.13, 95% CI: 0.45 to 1.81, t 3.56, *p* = 0.001). This result indicates the presence of substantial small-study effects, a hallmark of publication bias. Consistent with this finding, Begg and Mazumdar’s rank correlation test also demonstrated significant evidence of bias (Kendall’s *τ* = 0.39, *p* = 0.002), indicating a strong correlation between study effect sizes and their variances.

Despite the clear statistical evidence of publication bias, its impact on the overall conclusion is likely minimal. The primary meta-analysis yielded a robust and highly significant summary effect (OR = 3.87, 95% CI: 3.06 to 4.88, *p* < 0.00001). The strength of this association is critical for two reasons. First, the lower bound of the 95% confidence interval (3.06) remains substantially greater than 1, indicating a persistent and strong effect even at the most conservative estimate. Second, the magnitude of the observed effect is so large that it is highly improbable for unpublished null studies to completely negate this finding. The consistent direction and significance across the majority of the included studies further bolster the conclusion that the identified association is genuine and not solely an artifact of selective publication.

In summary, while publication bias cannot be ruled out and may have led to a modest overestimation of the effect size, the convergent evidence from a large, strong, and highly consistent effect suggests that the fundamental conclusion of the meta-analysis is robust.

### 3.5 Reliability of the evidence

**Figure 20:**
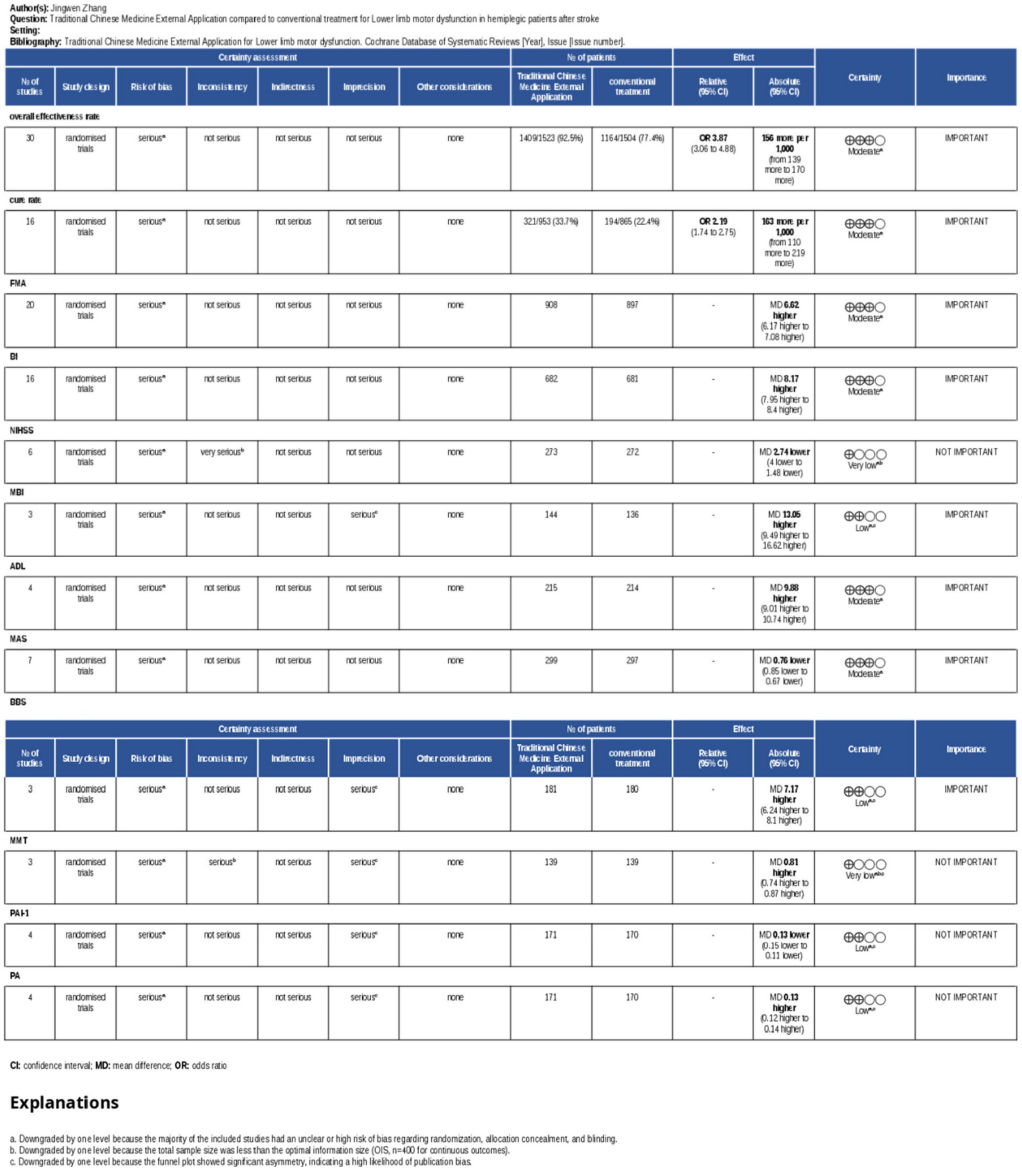
Grading of quality of evidence of outcome metrics.

The GRADE approach was employed to conduct a systematic evaluation of the evidence quality for each outcome indicator. Ultimately, the evidence quality grades of each outcome ranged from moderate to very low, which are specifically summarized in the Summary of Findings table (Supplementary Table S1) and visually presented in Figure 20.

The key determinants leading to the downgrading of evidence quality included: risk of bias (such as limitations in randomization or blinding implementation), inconsistency (especially the unexplainable heterogeneity in NIHSS and MMT scores), imprecision (the total sample size of some continuous outcomes was less than the optimal information sample size of 400 cases), and potential publication bias (suggested by the asymmetry of the funnel plot). The specific criteria and detailed judgment bases for the GRADE assessment were fully documented in the Summary of Findings table (Supplementary Table S1).

## 4 Discussion

### 4.1 Strengths and limitations

This systematic review and meta-analysis, incorporating 43 RCTs and 4,186 patients, provides a comprehensive quantitative synthesis supporting the efficacy of TCM external therapies for improving lower limb motor function in post-stroke hemiplegia. Our findings demonstrate that adjunctive TCM external therapy yields significant improvements across multiple functional domains, including motor recovery (FMA), activities of daily living (Barthel Index, MBI, and ADL), neurological impairment (NIHSS), muscle spasticity (MAS), balance (BBS), and serum biomarkers of fibrinolysis (PAI-1 and t-PA). This pattern of multi-faceted improvement aligns with the growing recognition of integrative medicine approaches in stroke rehabilitation, which aim to synergize conventional and complementary therapeutic strategies to optimize patient outcomes (48). Several limitations merit consideration. Methodological quality was often compromised by inadequate reporting of randomization, allocation concealment, and blinding, potentially introducing performance and detection bias. The general absence of long-term follow-up precludes conclusions on sustained benefits. Furthermore, while employing internationally recognized assessment scales, the predominant reliance on clinical evaluations introduces an inherent element of subjectivity. Future investigations would be strengthened by incorporating objective biomarkers and neurophysiological measures, such as quantitative gait analysis and motor-evoked potentials, to corroborate clinical findings (49). Funnel plot asymmetry suggests potential publication bias for the total effective rate, warranting cautious interpretation of effect magnitude. Notably, our subgroup analysis of NIHSS scores revealed that treatment duration is a critical factor influencing outcome consistency. Studies with treatment durations ≥28 days demonstrated perfect homogeneity (I^2^ = 0%), while those with shorter durations exhibited substantial heterogeneity (I^2^ = 95%). This finding provides empirical evidence for establishing a minimum 28-day treatment course in clinical practice to ensure consistent therapeutic effects, and may reflect the time required for neural plasticity and functional reorganization in stroke recovery.

### 4.2 Mechanism study

The consistent benefits across functional domains suggest that TCM external therapies operate through multi-targeted, synergistic mechanisms. While TCM philosophy emphasizes acupoint stimulation and meridian regulation of “Qi” and blood (50, 51), contemporary research points to several evidence-based pathways. The transdermal delivery of bioactive compounds represents a fundamental initial step, facilitated by the distinctive permeability characteristics of both skin and acupoints, which may function as specialized portals for enhanced drug absorption and neural signal transduction (52, 53).

1. Enhancement of Local Hemodynamics and Microcirculation: A key mechanism involves improved regional blood flow. Thermotherapeutic applications (e.g., hot ironing and fumigation) combined with vasoactive phytochemical constituents (e.g., tanshinone IIA from *Salvia miltiorrhiza*) induce substantial vasodilation, thereby augmenting perfusion in paretic limbs (54). This optimized microcirculatory milieu is critical for enhancing oxygen and nutrient delivery while facilitating the clearance of metabolic waste, creating a conducive environment for the repair of neural and muscular tissues. Such peripheral vascular enhancement is a well-established facilitator of neurorehabilitation (55).
2. Modulation of Neural Plasticity and Cortical Reorganization: Motor recovery post-stroke relies on neuroplasticity—the central nervous system’s capacity for functional and structural adaptation. Evidence indicates that the rich somatosensory stimulation provided by TCM external therapies, particularly through acupoint stimulation, generates potent afferent signals that promote this adaptive reorganization. The underlying mechanisms may involve the upregulation of key neurotrophic factors (e.g., BDNF), modulation of neurotransmitter systems, and the functional reorganization of cortical motor maps, collectively refining motor programming and execution (56, 57). The field of neuromodulation underscores the importance of such targeted sensory inputs in driving beneficial central nervous system adaptations (49).
3. Mitigation of Spasticity and Inflammatory Pathways: Managing spasticity and neuroinflammation is crucial for functional recovery. The demonstrated antispasmodic and anti-inflammatory properties of numerous external application herbs (e.g., *Phellodendron amurense*) enable modulation of spinal reflex circuits and suppression of pro-inflammatory cytokine cascades (58, 59). The reduction of muscle hypertonia directly improves motor performance, while the concurrent attenuation of neuroinflammation and peripheral edema fosters a more permissive microenvironment for neural repair and synaptic plasticity. The well-documented detrimental role of persistent inflammation in stroke recovery highlights the therapeutic value of these anti-inflammatory strategies (60).
4. Temporal Dynamics of Therapeutic Effects and the Critical Treatment Window: Beyond the immediate pharmacological and physiological actions, the temporal dimension of therapy is crucial for achieving stable and reproducible outcomes. Our meta-analysis revealed a striking pattern: studies implementing TCM external therapies for durations of ≥28 days demonstrated perfect homogeneity (I^2^ = 0%) in improving neurological deficits (NIHSS), whereas shorter regimens yielded highly inconsistent results. This 28-day threshold is not arbitrary but resonates profoundly with established neurobiological timelines for stroke recovery. It likely corresponds to the convergence of several critical, time-dependent restorative processes: First, it aligns with the period required for significant axonal sprouting and synaptic reorganization within peri-infarct regions, a process driven by the upregulation of growth-associated proteins and the formation of new neural connections (55). Second, it encompasses a key phase for cortical map reorganization and motor learning consolidation, where sustained afferent input from acupoint stimulation can effectively compete for cortical representation space and solidify new motor engrams (61). Finally, this window supports angiogenesis and blood–brain barrier restoration, which are essential for delivering metabolic support and creating a conducive microenvironment for neural repair (62). This temporal pattern underscores that the benefits of TCM external therapy extend beyond transient symptomatic relief, necessitating sustained intervention to co-evolve with the brain’s innate, yet slow, reparative processes. It argues for a paradigm shift from short-term symptom management to a commitment to adequate treatment duration, thereby ensuring the full engagement of neural plasticity to achieve lasting functional recovery.

In summary, the mechanistic framework of TCM external therapy likely involves a sophisticated interplay between peripheral actions (improved circulation, reduced spasticity, and inflammation) and central effects (enhanced neuroplasticity). This multi-level approach resonates with the holistic principles of TCM and addresses the complex pathophysiology of post-stroke recovery. While the existing evidence is promising, further rigorous investigations employing advanced pharmacological and neurophysiological techniques are essential to precisely delineate the molecular targets and signaling pathways involved, thereby bridging traditional practice with contemporary evidence-based medicine.

### 4.3 Prospects

Clinical evidence from this meta-analysis supports the potential of topical TCM for improving lower limb motor function in post-stroke hemiplegia. To address current limitations and advance the field, future research should prioritize.

1. Elucidating Underlying Mechanisms: Move beyond macroscopic efficacy to investigate mechanisms using molecular biology, proteomics, and multimodal neuroimaging (e.g., fMRI and DTI) to identify specific molecular targets and intracellular signaling pathways. Employing advanced techniques from molecular biology, proteomics, and multimodal neuroimaging (e.g., fMRI and DTI) is crucial to identify specific molecular targets and intracellular signaling pathways involved in promoting neurovascular unit repair, modulating neuroinflammation, and inducing cortical reorganization. This is essential for translating the empirical descriptions of TCM into a biological language comprehensible to modern medicine.
2. Standardizing Interventions and Exploring Personalization: A critical bottleneck for clinical application is the lack of standardized protocols. Our finding that treatment durations ≥28 days yield consistent results (I^2^ = 0%) provides empirical support for establishing this as a minimum treatment course. There is an urgent need to establish unified operational procedures, encompassing core herbal formulations, solvent concentrations, single-session duration, treatment frequency, and total course of treatment (with 28 days as a benchmark), through rigorous dose–response studies and Delphi-based expert consensus.
3. Exploring Integrated TCM-Western Rehabilitation Models: Investigate deep integration of TCM and Western rehabilitation theories. The combination of topical TCM (e.g., fumigation and hot ironing) and modern rehabilitation techniques (e.g., robot-assisted gait training, task-oriented training) holds promise for generating synergistic effects based on complementary mechanisms. Specifically, topical TCM may act peripherally by improving local blood circulation, reducing tissue edema, and modulating muscle tension, thereby creating an optimal “physiological environment” for nerve repair and motor relearning. In contrast, modern rehabilitation primarily acts centrally, promoting brain cortical functional reorganization and motor program reconstruction through high-intensity, repetitive, task-specific stimulation. Therefore, future research should extend beyond verifying “whether the combination is effective” to investigating the “scientific principles” and “optimal models” of integration. For instance, well-designed crossover studies could examine whether a “physiological pre-conditioning” with topical TCM before motor training enhances motor learning efficiency and functional gains more than simply extending training duration. This mechanism—complementary sequential treatment model—has the potential to break through the current plateau in rehabilitation therapy, truly achieving a ‘1 + 1 > 2’ synergistic effect, and providing high-grade evidence for constructing an integrated stroke rehabilitation framework with Chinese characteristics.
4. Generating High-Quality Evidence with Methodological Rigor: The reliability of conclusions depends on the quality of primary studies. We strongly advocate for large-sample, multi-center, randomized controlled trials that adhere to the CONSORT reporting guidelines and implement rigorous blinding. These trials should be prospectively registered on clinical trial platforms and prioritize clinically meaningful objective functional outcomes as primary endpoints.

Finally, to facilitate the transition of topical TCM from an experience-based to an evidence-based practice and to promote its standardized application internationally, a globally recognized standardization system must be established. This system should encompass three core dimensions: First, the standardization of technical operations, i.e., developing and disseminating detailed, operable clinical practice guidelines based on the best available evidence, clearly defining indications, contraindications, operational procedures, key parameters (e.g., temperature, duration), and adverse event management protocols for different external techniques. Second, the standardization of quality control requires establishing a whole-process quality control standard from herb cultivation, processing, and preparation production to final product stability, ensuring the uniformity and replicability of the interventions. Third, the standardization of safety evaluation and regulation necessitates the establishment of systematic systems for monitoring, reporting, and assessing adverse reactions, and forming review standards that can be understood and accepted by regulatory agencies worldwide. Through these efforts, we can not only maximize patient safety and the stability of clinical efficacy but also provide a clear, scientific “identity language” for topical TCM. This will help transcend cultural and traditional cognitive barriers, clearing the fundamental obstacles for its inclusion in international mainstream clinical guidelines, insurance systems, and its safe, standardized application worldwide.

In conclusion, this meta-analysis provides consolidated evidence supporting the efficacy of topical TCM in improving lower limb motor function after stroke. To translate this potential into consistent, globally accepted clinical practice, future efforts must be prioritized along the key trajectories outlined above: elucidating mechanistic underpinnings, establishing standardized protocols, innovating integrated rehabilitation models, and generating robust clinical evidence. Through these concerted actions, topical TCM can evolve from an empirical practice into an evidence-based mainstay of post-stroke rehabilitation, ultimately contributing to improved functional recovery and quality of life for patients worldwide.

## Data Availability

The original contributions presented in the study are included in the article/Supplementary material, further inquiries can be directed to the corresponding author.
