# Supplementary material for: Meta-analysis of the efficacy of external application of Chinese medicine in the treatment of lower limb motor impairment in patients with post-stroke hemiplegia
Source: Front Neurol. 2025 Nov 28;16:1691805. doi: 10.3389/fneur.2025.1691805 (PMC12698439; doi:10.3389/fneur.2025.1691805)
Supplement: Supplementary file 1 [file Data_Sheet_1.PDF]

Summary of survey Results (SoF) table

Outcome Measures	Inclusion Number of Studies	Number of participants	Effect size (95% CI)	Evidence Level	Risk of bias	Inconsistency	Indirectness	Imprecision	Bias of publication	Summary of reasons for downgrade
Total efficiency	30	3027	OR = 3.87 (3.06, 4.88)	Medium	-1	0	0	0	-1	Risk of bias: most studies were insufficiently randomized and blinded. Publication bias: Funnel plot asymmetry. Inconsistency: I^2=0%, no heterogeneity. Imprecision: CI narrow, did not cross the equivalence line, sample size >OIS.
recovery rate	16	1818	OR = 2.19 (1.74, 2.75)	Medium	-1	0	0	0	-1	Risk of bias: most studies were insufficiently randomized and blinded. Publication bias: Funnel plot asymmetry.

Outcome Measures	Inclusion Number of Studies	Number of participants	Effect size (95% CI)	Evidence Level	Risk of bias	Inconsistency	Indirectness	Imprecision	Bias of publication	Summary of reasons for downgrade
										Inconsistency: I^2=0%, no heterogeneity.<br>Imprecision: CI narrow, did not cross the equivalence line, sample size >OIS.
FMA score	20	1805	MD = 6.62 (6.17, 7.08)	Medium	-1	0	0	0	-1	Risk of bias: Most studies had a moderate risk of bias.<br>Publication bias: Overall, there was publication bias.<br>Inconsistency: I^2=49%, moderate heterogeneity but consistent direction.<br>Imprecision: CI narrow, did not cross the equivalence line, sample size >OIS.

Outcome Measures	Inclusion Number of Studies	Number of participants	Effect size (95% CI)	Evidence Level	Risk of bias	Inconsistency	Indirectness	Imprecision	Bias of publication	Summary of reasons for downgrade
Barthel index	16	1363	MD = 8.17 (7.95, 8.40)	Medium	-1	0	0	0	-1	Risk of bias: Most studies had a moderate risk of bias.<br>Publication bias: Overall, there was publication bias.<br>Inconsistency: I^2=13%, low heterogeneity.<br>Imprecision: CI extremely narrow, sample size >OIS.
Modified Barthel Index	3	280	MD = 13.05 (9.49, 16.62)	low	-1	0	0	-1	-1	Risk of bias: Most studies had a moderate risk of bias.<br>Imprecision: Sample size <400 (OIS).<br>Publication bias: There was overall publication bias.<br>Inconsistency: I^2=0%, no heterogeneity.

Outcome Measures	Inclusion Number of Studies	Number of participants	Effect size (95% CI)	Evidence Level	Risk of bias	Inconsistency	Indirectness	Imprecision	Bias of publication	Summary of reasons for downgrade
ADL score	4	429	MD = 9.88 (9.01, 10.74)	Medium	-1	0	0	0	-1	Risk of bias: Most studies had a moderate risk of bias. Publication bias: Overall, there was publication bias. Inconsistency: I^2=12%, low heterogeneity. Imprecision: narrow CI, sample size >OIS.
NIHSS score	6 (4)*	545	MD = -2.72 (-3.28, -2.16)	Very low	-1	-2	0	0	-1	Risk of bias: Most studies had a moderate risk of bias. Inconsistency: initial I^2=83%, high heterogeneity, need sensitivity analysis to address. Publication bias: There was overall publication bias. Imprecision: the CI was narrow and did not cross the equivalence line.

Outcome Measures	Inclusion Number of Studies	Number of participants	Effect size (95% CI)	Evidence Level	Risk of bias	Inconsistency	Indirectness	Imprecision	Bias of publication	Summary of reasons for downgrade
MAS score	7	596	MD = -0.76 (-0.85, -0.67)	Medium	-1	0	0	0	-1	Risk of bias: Most studies had a moderate risk of bias.<br>Publication bias: Overall, there was publication bias.<br>Inconsistency: I^2=0%, no heterogeneity.<br>Imprecision: narrow CI, sample size >OIS
BBS score	3	361	MD = 7.17 (6.24, 8.10)	low	-1	0	0	-1	-1	Risk of bias: Most studies had a moderate risk of bias.<br>Imprecision: Sample size <400 (OIS).<br>Publication bias: There was overall publication bias.

Outcome Measures	Inclusion Number of Studies	Number of participants	Effect size (95% CI)	Evidence Level	Risk of bias	Inconsistency	Indirectness	Imprecision	Bias of publication	Summary of reasons for downgrade
										Inconsistency: I^2=8%, low heterogeneity.
MMT score	3 (2)*	278	MD = 0.78 (0.73, 0.83)	Very low	-1	-1	0	-1	-1	Risk of bias: Most studies had a moderate risk of bias. Inconsistency: initial I^2=53%, moderate heterogeneity, a sensitivity analysis was required to address. Imprecision: sample size <400 (OIS). Publication bias: There was overall publication bias.
PAI-1 levels	4	341	MD = -0.13 (-0.15, -0.11)	low	-1	0	0	-1	-1	Risk of bias: Most studies had a moderate risk of bias. Imprecision: Sample size <400 (OIS).

Outcome Measures	Inclusion Number of Studies	Number of participants	Effect size (95% CI)	Evidence Level	Risk of bias	Inconsistency	Indirectness	Imprecision	Bias of publication	Summary of reasons for downgrade
										Publication bias: There was overall publication bias.<br>Inconsistency: I^2=0%, no heterogeneity.
t-PA levels	4	341	MD = 0.13 (0.12, 0.14)	low	-1	0	0	-1	-1	Risk of bias: Most studies had a moderate risk of bias.<br>Imprecision: Sample size <400 (OIS).<br>Publication bias: There was overall publication bias.<br>Inconsistency: I^2=0%, no heterogeneity.

Notes:

1. (n)* : For NIHSS and MMT scores, the final effect size is based on a subset of studies after sensitivity analysis.
2. The starting point for the level of evidence was "high" (because all were RCTS).
3. OIS (optimal informative sample size) : The continuous variable was set at 400.

4. Downgrade factor sign: -1 (1 level down), -2 (2 levels down), 0 (no downgrade).
