# Supplementary material for: Meta-analysis of the efficacy of external application of Chinese medicine in the treatment of lower limb motor impairment in patients with post-stroke hemiplegia
Source: Front Neurol. 2025 Nov 28;16:1691805. doi: 10.3389/fneur.2025.1691805 (PMC12698439; doi:10.3389/fneur.2025.1691805)
Supplement: Supplementary file 2 [file Data_Sheet_2.DOCX]

**Author(s):** Jingwen Zhang

**Question:** Traditional Chinese Medicine External Application compared to conventional treatment for Lower limb motor dysfunction in hemiplegic patients after stroke

**Setting:**

**Bibliography:** Traditional Chinese Medicine External Application for Lower limb motor dysfunction. Cochrane Database of Systematic Reviews [Year], Issue [Issue number].

| **Certainty assessment** | | | | | | | **№ of patients** | | **Effect** | | **Certainty** | **Importance** |
| --- | --- | --- | --- | --- | --- | --- | --- | --- | --- | --- | --- | --- |
| **№ of studies** | **Study design** | **Risk of bias** | **Inconsistency** | **Indirectness** | **Imprecision** | **Other considerations** | **Traditional Chinese Medicine External Application** | **conventional treatment** | **Relative (95% CI)** | **Absolute (95% CI)** |  |  |
| **overall effectiveness rate** | | | | | | | | | | | | |
| 30 | randomised trials | serious^a^ | not serious | not serious | not serious | none | 1409/1523 (92.5%) | 1164/1504 (77.4%) | **OR 3.87** (3.06 to 4.88) | **156 more per 1,000** (from 139 more to 170 more) | ⨁⨁⨁◯ Moderate^a^ | IMPORTANT |
| **cure rate** | | | | | | | | | | | | |
| 16 | randomised trials | serious^a^ | not serious | not serious | not serious | none | 321/953 (33.7%) | 194/865 (22.4%) | **OR 2.19** (1.74 to 2.75) | **163 more per 1,000** (from 110 more to 219 more) | ⨁⨁⨁◯ Moderate^a^ | IMPORTANT |
| **FMA** | | | | | | | | | | | | |
| 20 | randomised trials | serious^a^ | not serious | not serious | not serious | none | 908 | 897 | - | MD **6.62 higher** (6.17 higher to 7.08 higher) | ⨁⨁⨁◯ Moderate^a^ | IMPORTANT |
| **BI** | | | | | | | | | | | | |
| 16 | randomised trials | serious^a^ | not serious | not serious | not serious | none | 682 | 681 | - | MD **8.17 higher** (7.95 higher to 8.4 higher) | ⨁⨁⨁◯ Moderate^a^ | IMPORTANT |
| **NIHSS** | | | | | | | | | | | | |
| 6 | randomised trials | serious^a^ | very serious^b^ | not serious | not serious | none | 273 | 272 | - | MD **2.74 lower** (4 lower to 1.48 lower) | ⨁◯◯◯ Very low^a,b^ | NOT IMPORTANT |
| **MBI** | | | | | | | | | | | | |
| 3 | randomised trials | serious^a^ | not serious | not serious | serious^c^ | none | 144 | 136 | - | MD **13.05 higher** (9.49 higher to 16.62 higher) | ⨁⨁◯◯ Low^a,c^ | IMPORTANT |
| **ADL** | | | | | | | | | | | | |
| 4 | randomised trials | serious^a^ | not serious | not serious | not serious | none | 215 | 214 | - | MD **9.88 higher** (9.01 higher to 10.74 higher) | ⨁⨁⨁◯ Moderate^a^ | IMPORTANT |
| **MAS** | | | | | | | | | | | | |
| 7 | randomised trials | serious^a^ | not serious | not serious | not serious | none | 299 | 297 | - | MD **0.76 lower** (0.85 lower to 0.67 lower) | ⨁⨁⨁◯ Moderate^a^ | IMPORTANT |
| **BBS** | | | | | | | | | | | | |
| 3 | randomised trials | serious^a^ | not serious | not serious | serious^c^ | none | 181 | 180 | - | MD **7.17 higher** (6.24 higher to 8.1 higher) | ⨁⨁◯◯ Low^a,c^ | IMPORTANT |
| **MMT** | | | | | | | | | | | | |
| 3 | randomised trials | serious^a^ | serious^b^ | not serious | serious^c^ | none | 139 | 139 | - | MD **0.81 higher** (0.74 higher to 0.87 higher) | ⨁◯◯◯ Very low^a,b,c^ | NOT IMPORTANT |
| **PAI-1** | | | | | | | | | | | | |
| 4 | randomised trials | serious^a^ | not serious | not serious | serious^c^ | none | 171 | 170 | - | MD **0.13 lower** (0.15 lower to 0.11 lower) | ⨁⨁◯◯ Low^a,c^ | NOT IMPORTANT |
| **PA** | | | | | | | | | | | | |
| 4 | randomised trials | serious^a^ | not serious | not serious | serious^c^ | none | 171 | 170 | - | MD **0.13 higher** (0.12 higher to 0.14 higher) | ⨁⨁◯◯ Low^a,c^ | NOT IMPORTANT |

**CI:** confidence interval; **MD:** mean difference; **OR:** odds ratio

#### Explanations

a. Downgraded by one level because the majority of the included studies had an unclear or high risk of bias regarding randomization, allocation concealment, and blinding.

b. Downgraded by one level because the total sample size was less than the optimal information size (OIS, n=400 for continuous outcomes).

c. Downgraded by one level because the funnel plot showed significant asymmetry, indicating a high likelihood of publication bias.
